# Structural basis for Fc receptor recognition of immunoglobulin M

**DOI:** 10.1038/s41594-023-00985-x

**Published:** 2023-04-24

**Authors:** Qu Chen, Rajesh P. Menon, Laura Masino, Pavel Tolar, Peter B. Rosenthal

**Affiliations:** 1 Structural Biology Science Technology Platform, The Francis Crick Institute, 1 Midland Road, London, NW1 1AT, UK; 2 Immune Receptor Activation Laboratory, The Francis Crick Institute, 1 Midland Road, London, NW1 1AT, UK; 3 Institute of Immunity and Transplantation, University College London, Rowland Hill Street, London, NW3 2PP, UK; 4 Structural Biology of Cells and Viruses Laboratory, The Francis Crick Institute, 1 Midland Road, London, NW1 1AT, UK

## Abstract

Immunoglobulin Fc receptors are cell surface transmembrane proteins that bind to the Fc constant region of antibodies and play critical roles in regulating immune responses by activation of immune cells, clearance of immune complexes, and regulation of antibody production. FcμR is the IgM antibody isotype-specific Fc receptor involved in the survival and activation of B cells. Here we reveal eight binding sites for the human FcμR immunoglobulin (Ig) domain on the IgM pentamer by cryo-EM. One of the sites overlaps with the binding site for the transcytosis receptor pIgR, but a different mode of FcμR binding explains its antibody isotype specificity. Variation in FcμR binding sites and their occupancy reflects the asymmetry of the IgM pentameric core and the versatility of FcμR binding. The complex explains engagement with polymeric serum IgM and the monomeric IgM B cell receptor.

## Introduction

Immunoglobulin Fc receptors are effector molecules expressed on the surface of immune cells, which can generate a wide range of protective functions crucial in immune responses after engaging with the Fc domains of the immunoglobulins. FcμR (historically also called TOSO or FAIM3) is a high-affinity Fc receptor specific for IgM^1^. Although IgM is a primordial Ig isotype present in all vertebrates, FcμR has a relatively late appearance during early mammalian evolution^2^. Full-length human FcμR has 390 amino acids (aa). 251 aa are extracellular, including a 17-aa signal peptide, a 107-aa immunoglobulin (Ig)-like domain, and a 127-aa stalk. The rest of the receptor consists of a 21-aa transmembrane region and a long 118-aa cytoplasmic tail at the C-terminus^3^. The Ig-like domain is responsible for ligand binding^3^ and shares about 40% sequence identity with the first Ig-like domain of the polymeric immunoglobulin receptor (pIgR-D1), which is encoded by a gene located in the same chromosomal region in mammals^2^. However, rather than binding to both polymeric IgM and IgA as pIgR does^4–7^, FcμR exclusively binds to IgM^8^, indicating its specific binding mechanisms as well as functional roles. Potential residues responsible for the binding between IgM and FcμR have been proposed but the structure of the FcμR/IgM complex is currently unknown^9–11^. In this study, we image a complex of the ectodomain of human FcμR and the IgM-Fc pentameric core using single-particle cryogenic electron microscopy (cryo-EM). The analysis reveals multiple binding sites for the N-terminal Ig domain of FcμR on the Cμ4 domain dimers within the IgM constant region and provides a framework for understanding FcμR’s role in immunoglobulin recognition and signalling.

## Results

### Multiple binding sites for FcμR on the IgM pentamer

**Extended Data Fig. 1:**
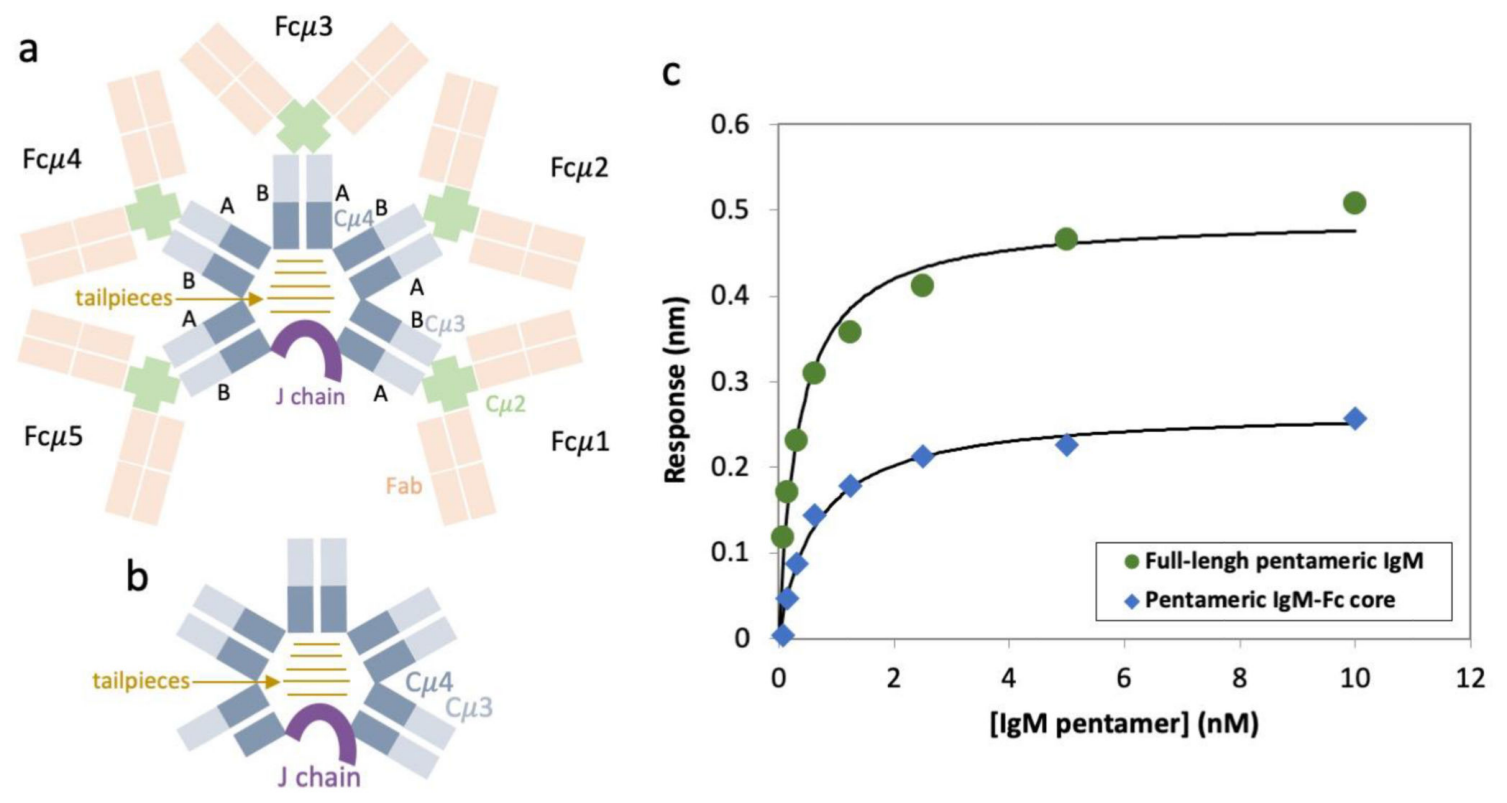
Binding of Full-length IgM and IgM-Fc core to surface-immobilised FcμR. (a) Structure schematic for full-length IgM. (b) Structure schematic for proteolysed IgM-Fc core. (c) Binding of full-length (green) and proteolysed IgM-Fc core (blue) to FcμR monitored by Bio-Layer Interferometry (BLI). Representative data sets for each form of IgM are shown. Instrument response values are plotted against IgM concentration. Fitting curves are shown as black lines. The apparent equilibrium dissociation constants (K_d_) for full-length IgM and IgM-Fc core are 0.3 ± 0.1 nM and 0.7 ± 0.1 nM respectively. Technical replicates gave values of 0.4 ± 0.1 nM and 0.7 ± 0.1 nM respectively. Raw data for the plot and the technical replicates are provided in the Source Data file.

**Fig. 1:**
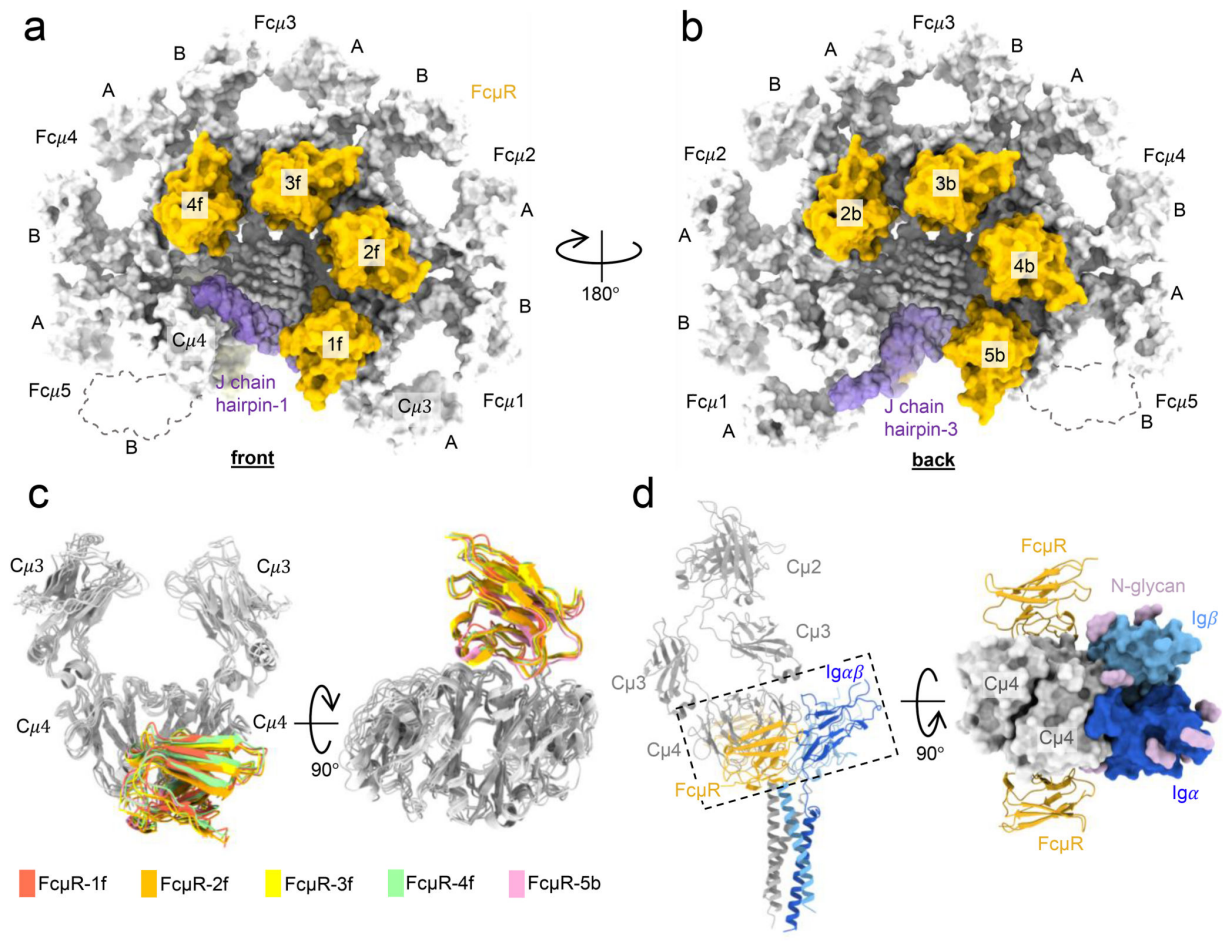
Multivalent binding of FcμR to pentameric IgM. (a-b) Structure of the human FcμR/IgM-Fc complex showing four FcμR binding at each side (a, front; b, back) of the IgM-Fc platform. Cμ3, Cμ4 and tailpieces of IgM in grey, and FcμR in yellow, with the names of the positions labelled on top of the domains. J chain in purple, with the hairpin-1 located at the front of IgM (a), while the hairpin-3 located at the back (b). The Cμ3-B domain in subunit Fcμ5, which is cleaved by proteolysis, is shown in dashed contour. (c) Superposition of subunit Fcμ1 to Fcμ5 aligned at Cμ4 domains showing the same binding position shared among the subunits. Taking FcμR-3f as reference, the backbone root mean squared deviation (RMSD) of FcμR-1f, FcμR-2f, FcμR-4f, and FcμR-5b is 2.6 Å, 1.4 Å, 1.6 Å, and 1.7 Å. (d) Overlay of the IgM-BCR (pdb id 7XQ8) and a subunit of IgM pentamer with two FcμR bound at both sides, aligned at the Cμ4 domains. Right panel, the top view of the region highlighted in the dashed box in the left panel shows no steric clash or interactions between FcμR and Ig*αβ* (in blue) on either side.

We obtained pentameric IgM-Fc core containing Cμ4, Cμ3 and the J chain by trypsin treatment of the full-length IgM^12^ (Extended Data Fig. 1a-b). We then measured the binding of the full-length IgM and the IgM-Fc core to surface-immobilised FcμR using bio-layer interferometry (BLI) and found them both to have similar subnanomolar affinity (Extended Data Fig. 1c). We therefore performed single particle cryo-EM on complexes of FcμR and the IgM-Fc core because it lacks the hinge and mobile antigen binding domains which complicate structural analysis. We resolved only the Ig domain of FcμR with the remaining ectodomain density appearing disordered at locations distal to the IgM binding site. Fig. 1a-b demonstrates the overall architecture of the multivalent engagement of the IgM pentamer by FcμR molecules. The IgM-Fc core closely resembles previously described pentameric IgM structures^4,6,13^, consisting of Cμ3 and Cμ4 domains, assembled at the extended C-terminal tailpieces with the J chain (Fig. 1a-b).The single copy of the J chain, which occupies the position of the sixth Fcμ subunits present in IgM hexamers, breaks the symmetry of the molecule and distinguishes the two sides of the IgM-Fc core. In this study, the side with the hairpin-1 loop of the J chain is defined as the front (Fig. 1a), and the opposite side with the hairpin-3 loop is defined as the back side of IgM (Fig. 1b). One of the Cμ3 domains, Cμ3-5B (the Cμ3 domain in Fcμ5B chain, represented by the dashed contours in Fig. 1a-b), is absent in the cryo-EM density map, indicating trypsin cleavage between Cμ3-5B and Cμ4-5B when producing the IgM-Fc core from full-length IgM molecules. This asymmetrical proteolysis among the Fcμ subunits is probably due to the asymmetry of IgM. Both Cμ3-5B domain and Cμ3-1A domain in Fcμ1A chain are next to the gap and therefore lack inter-subunit disulfide bonds at Cys414. However, Cμ3-1A domain directly interacts with the hairpin-3 loop of the J chain which slightly changes its orientation and stabilises it^13^. Cμ3-5B may be thus uniquely sensitive to proteolysis. The absence of the Cμ3-5B domain nevertheless does not interfere with the FcμR binding at subunit Fcμ5, as FcμR only recognises Cμ4 domains.

**Extended Data Fig. 2:**
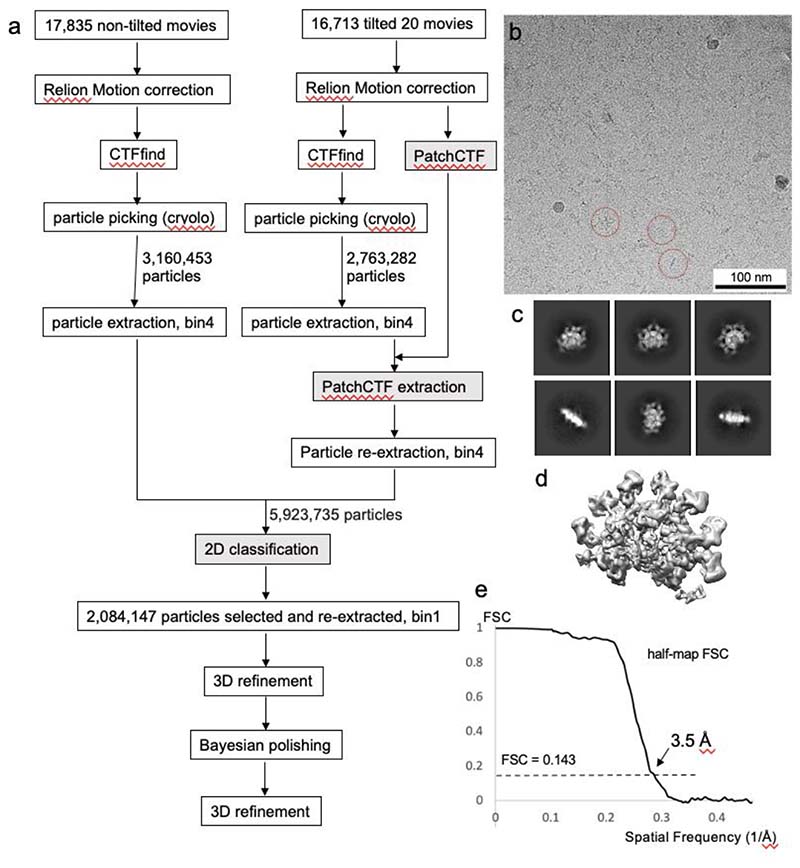
Single particle analysis of FcμR/IgM-Fc. (a) Flow chart of the data processing for both non-tilted and tilted datasets. Steps are conducted in Relion 3.1 (clear box) or Cryosparc 3.2.0 (grey box). (b) A typical micrograph with three particles highlighted in red dotted circles. (c) Typical 2D classes of the complex. (d) 3D auto-refined map. (e) Half-map Fourier shell correlation (FSC) plot showing 3.5 Å global resolution.

**Extended Data Fig. 3:**
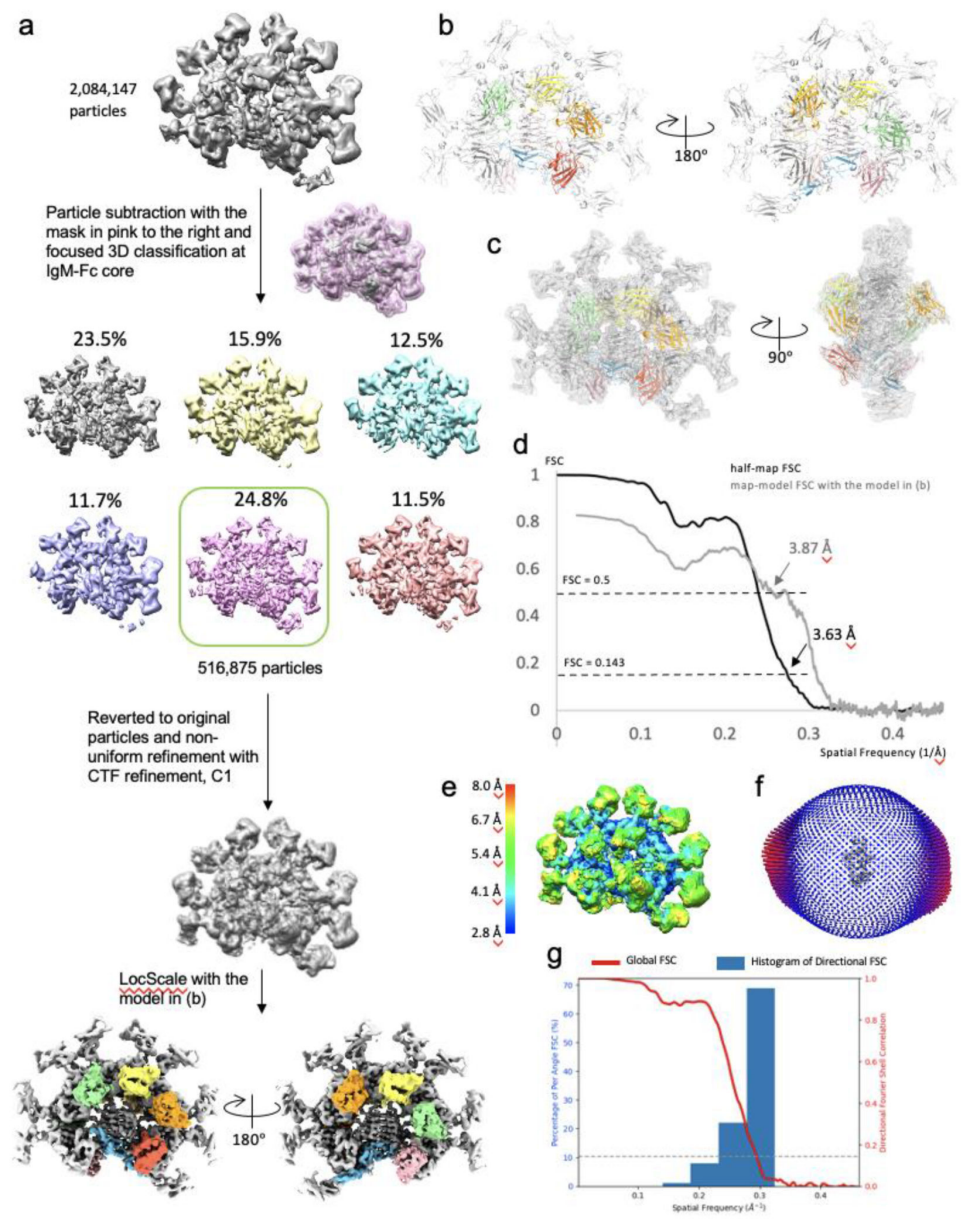
Cryo-EM structure of IgM-Fc/FcμR complex. (a) Particle subset selection by focused 3D classification at the IgM-Fc core and map refinement. (b) Front and side view of the complex model. IgM in grey, FcμR in rainboow colours. (c) Fitting of model and map shown in (a) and (b). (d) Fourier shell correlation (FSC) with 3.6 Å resolution at 0.143 cut-off and map-model FSC plot showing 3.9 Å resolution at 0.5 cut-off calculated with the model shown in (b) calculated in Phenix. (e) Local resolution of the refined map calculated in Cryosparc. (f) Eulerian angle distribution of the particles in the non-uniform refinement. (g) 3DFSC histogram calculated in Cryosparc of the refined map showing anisotropy between 3.2 Å -5.4 Å.

Eight FcμR binding sites are observed across the five Fcμ subunits of pentameric IgM, with four at the front of subunits Fcμ1 to Fcμ4 (positions 1f to 4f, Fig. 1a) and four at the back of subunits Fcμ2 to Fcμ5 (positions 2b to 5b, Fig. 1b, data processing workflow in Extended Data Fig. 2-3). Each FcμR is similarly positioned relative to the IgM subunit (Fig. 1c). Superposition of subunits of the IgM-FcμR complex on the IgM B-cell receptor (IgM-BCR) structure^14–16^, which contains a monomeric IgM (mIgM) identical to the subunits of the IgM pentamer at Cμ4 domains and two signalling chains Ig*αβ*, shows that mIgM-BCR can accommodate binding of two FcμR Ig domains (Fig. 1d).

### Variable occupancy of FcμR binding sites

An IgM pentamer has ten potential FcμR binding sites, but only eight are found to be occupied.The two FcμR-absent positions are the front of subunit Fcμ5 (position 5f) and the back of subunit Fcμ1 (position 1b) though extremely low occupancy at position 5f may be suggested by features near the noise level in unsharpened cryo-EM maps. The tip of the hairpin-1 region in the J chain may prevent the addition of an FcμR at position 5f by marginally blocking FcμR at Ser55-Thr57. Position 1b seems available for binding, but access to this site may be blocked by the hairpin-2 region in the J chain, a highly flexible loop containing 30 residues that remains unstructured in the cryo-EM map.

**Extended Data Fig. 4:**
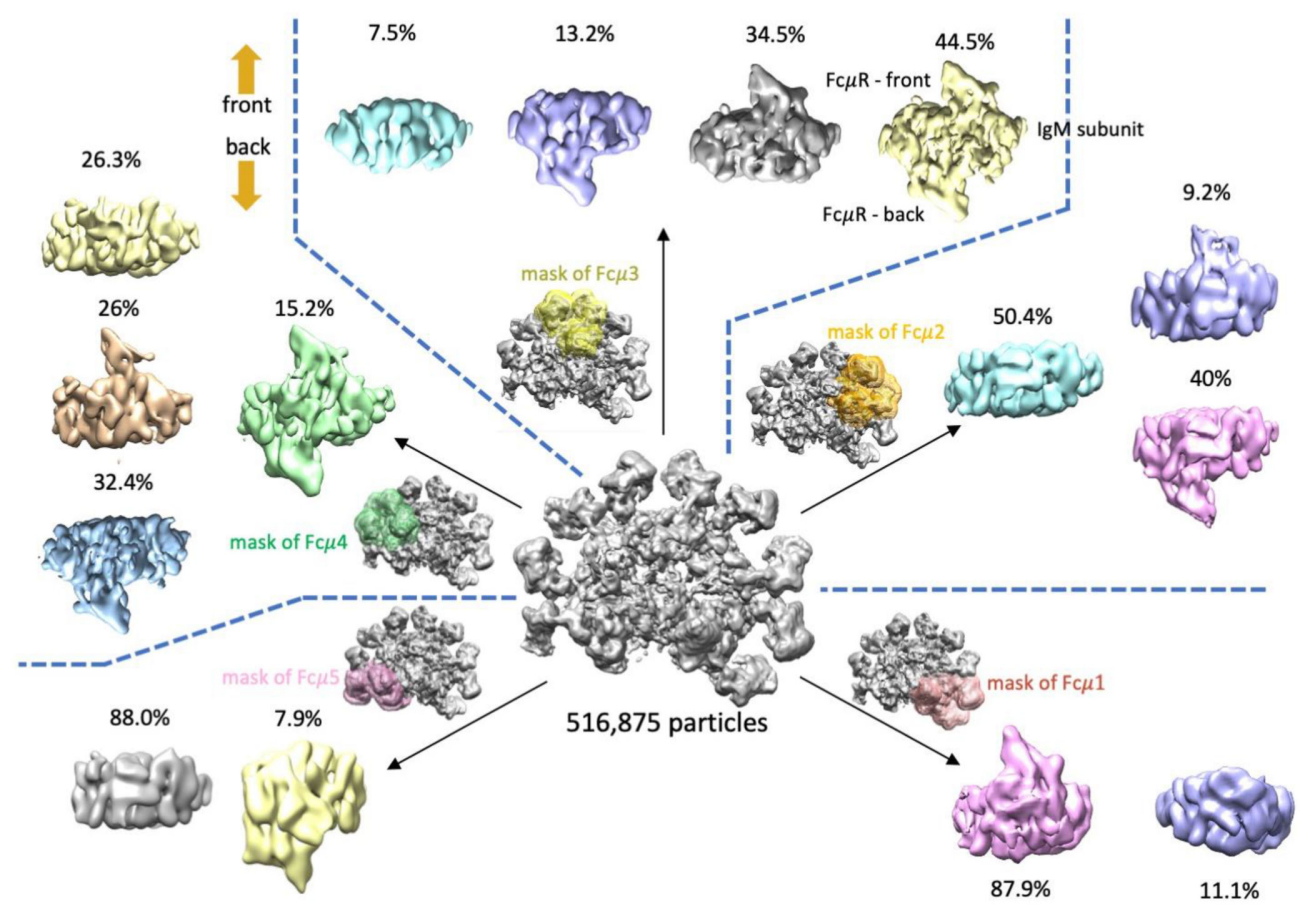
Maps of focused 3D classification at all IgM subunits Fcμ1 to Fcμ5 for quantification of FcμR occupancy at each subunit. The central EM density is the refined map shown in Extended Data Fig. 3, reconstructed with 516,875 particles. Mask used for focused 3D classification for each subunit (containing Cμ4 dimer, Cμ3 dimer and FcμR) is shown in a specific colour (subunit Fcμ1, red; subunit Fcμ2, orange; subunit Fcμ3, yellow; subunit Fcμ4, green; subunit Fcμ5, pink). The 3D classes show different FcμR binding states (at front, back, both or neither) at each subunit individually. The front and the back of IgM is shown at the top-left of the figure using the same definition described in the main text.

**Fig. 2:**
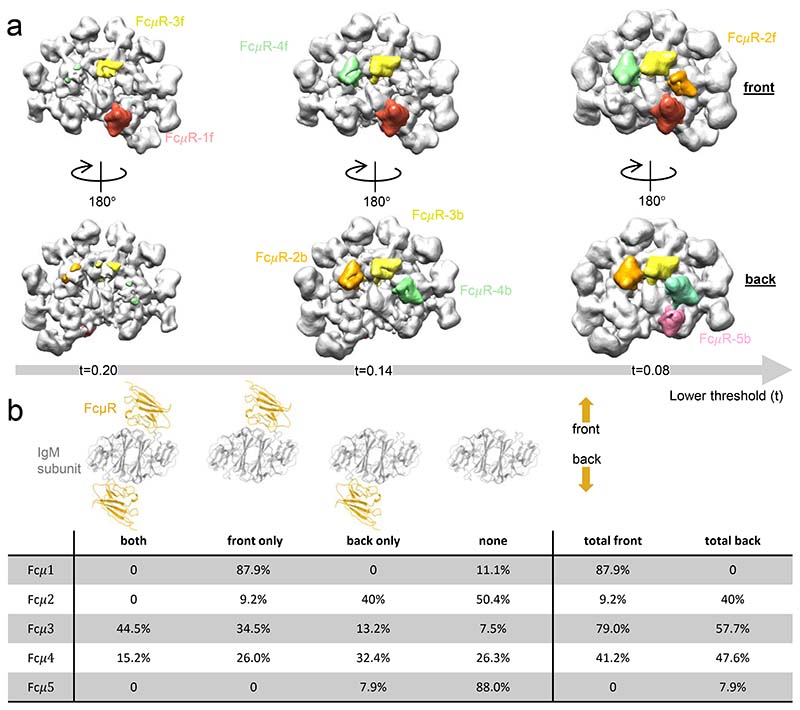
Different occupancies of FcμR among eight binding sites. (a) Gaussian-filtered (2 Å width) map (the non-uniform refined map in Extended Data Fig. 3a, before postprocessing) at three threshold levels showing the sequential appearance of FcμR densities from high to low occupancies. IgM in grey, FcμR in five colours representing the different binding positions. (b) Quantified occupancies of FcμR on individual subunits of IgM pentamer based on focused 3D classification described in Extended Data Fig. 4. The models on the top display the four FcμR binding states (bound to both sides, to front or back only, and none) on each IgM subunit classified by the focused 3D classification. The viewing direction of the models are from Cμ4 to Cμ3. The right two columns are the summed occupancies of FcμR binding at the front and the back of the subunits.

The eight binding sites on IgM pentamer are not equally occupied by FcμR. Lowering the threshold of the map (refined map in Extended Data Fig. 3) sequentially reveals the eight FcμR molecules from high to low densities, as shown in Fig. 2a, which is proportional to the occupancy of FcμR at each position. The concentration of FcμR used in forming complexes for cryo-EM imaging (1 μM) is similar to the reported K_d_ for FcμR binding to immobilised IgM^10^ and is therefore unlikely to be saturating for all binding sites. As a result, our maps indicate a diversity of FcμR affinity for the potential binding sites. Focused 3D classification at individual IgM subunits was also conducted to quantify the occupancies at each subunit (Extended Data Fig. 4), and the fractions of molecules with FcμR bound at either one, both or neither side of the IgM subunit are summarised in the table in Fig. 2b. The highest FcμR occupancies are at positions 1f and 3f (nearly 90%), followed by 2b-4b and 4f (40-60%), and with 2f and 5b the lowest (around 10%). The low occupancy at position 2f, which is sandwiched between the two most heavily occupied FcμR binding sites, may indicate a subtle steric hindrance between adjacent FcμR molecules.

### Recognition of IgM by FcμR Ig domain

**Extended Data Fig. 5:**
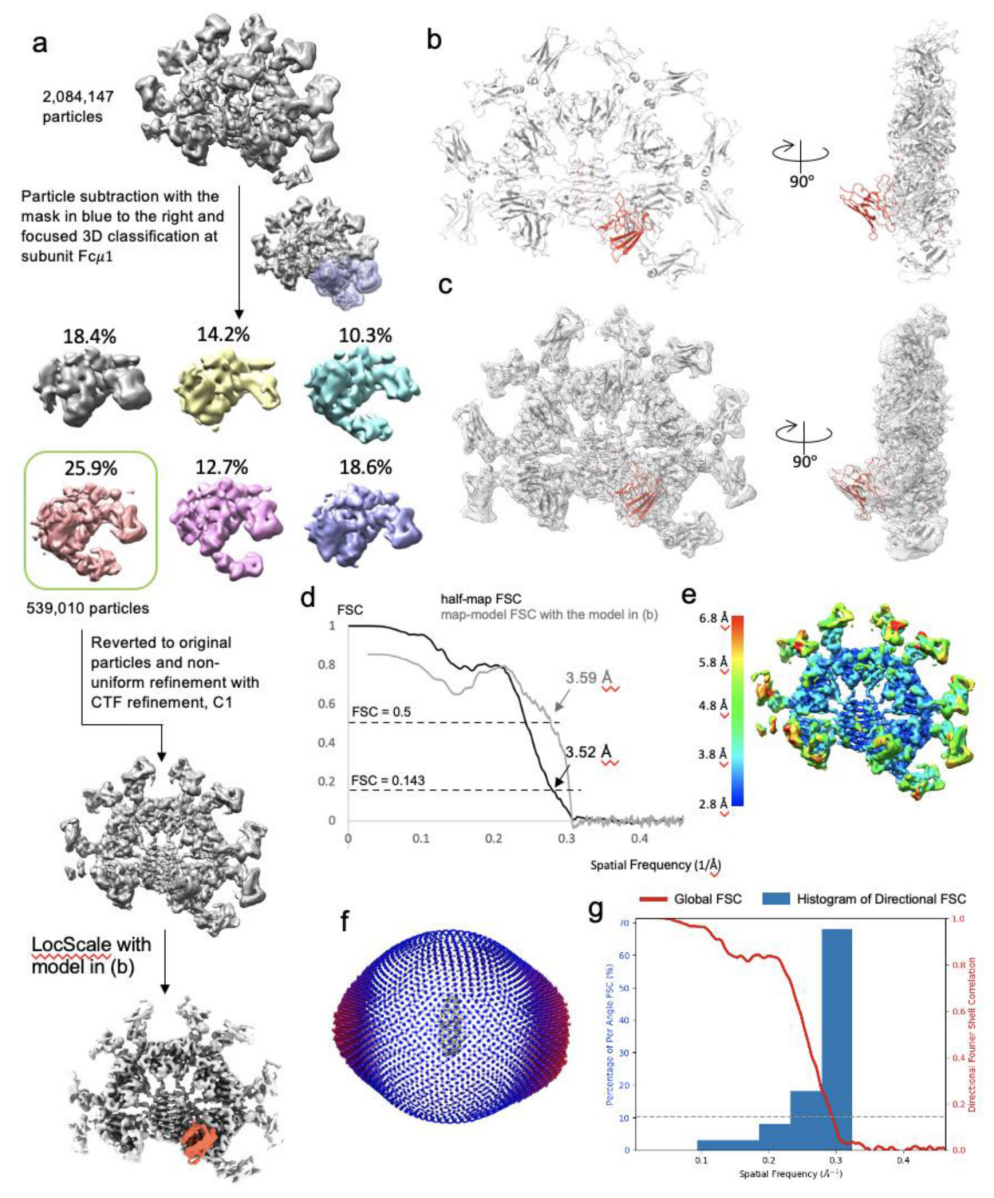
Cryo-EM structure of FcμR/IgM-Fc complex focused on subunit Fcμ1. (a) Particle subset selection by focused 3D classification at subunit Fcμ1 and map refinement. (b) Front and side view of the complex model. IgM in grey, FcμR in red. (c) Fitting of model and map shown in (a) and (b). (d) Fourier shell correlation (FSC) with 3.5 Å resolution at 0.143 cut-off and map-model FSC plot showing 3.6 Å resolution at 0.5 cut-off calculated in Phenix. (e) Local resolution of the refined map calculated in Cryosparc. (f) Eulerian angle distribution of the particles in the non-uniform refinement. (g) 3DFSC histogram of the refined map calculated in Cryosparc of the refined map showing anisotropy between 3.2 Å -7.6 Å.

**Extended Data Fig. 6:**
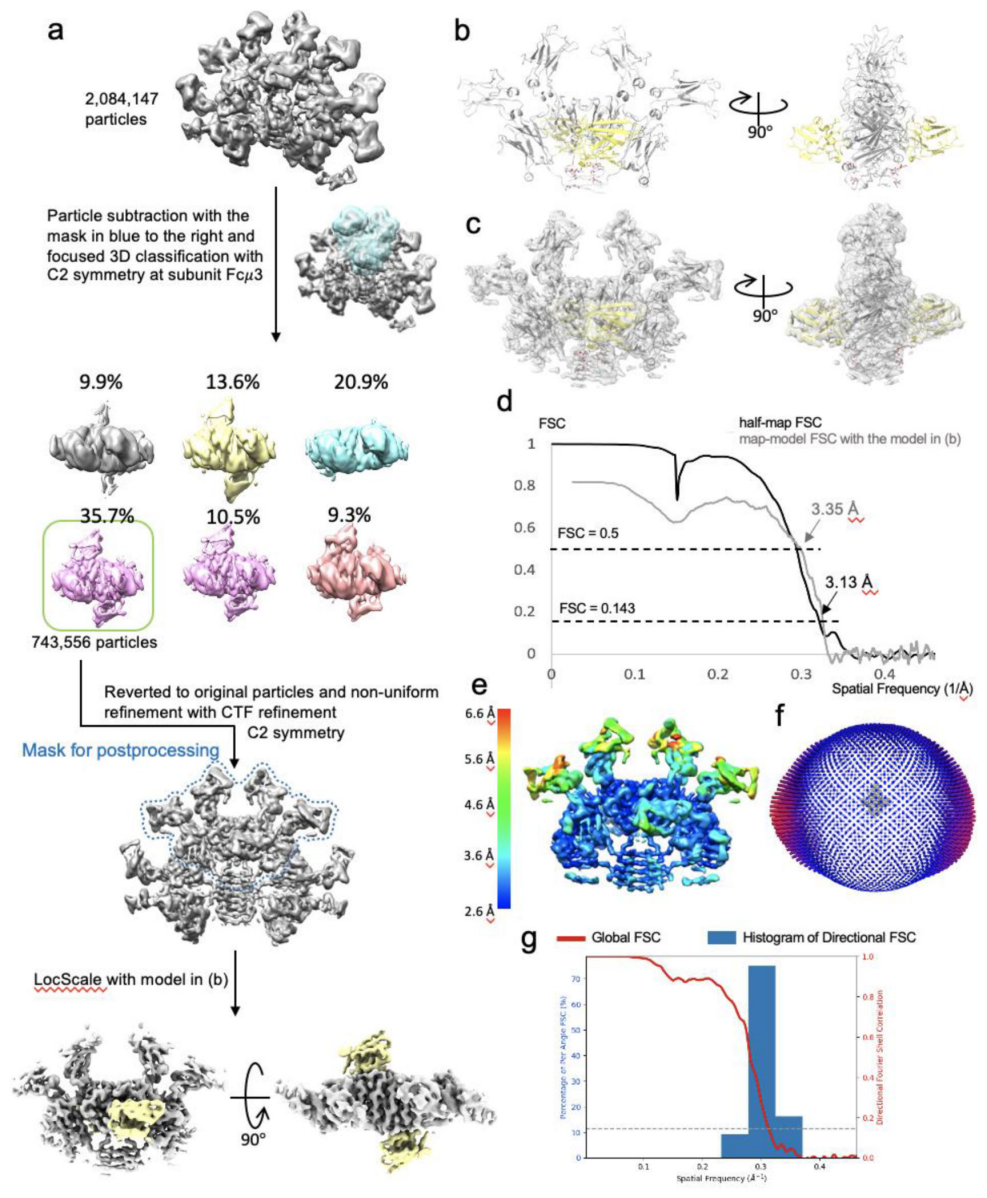
Cryo-EM structure of FcμR/IgM-Fc complex focused on subunit Fcμ3. (a) Particle subset selection by focused 3D classification at subunit Fcμ3 and map refinement. (b) Front and side view of the complex model. IgM in grey, FcμR in yellow. (c) Fitting of model and map shown in (a) and (b). (d) Fourier shell correlation (FSC) with 3.1 Å resolution at 0.143 cut-off and map-model FSC plot showing 3.3 Å resolution at 0.5 cut-off calculated in Phenix. (e) Local resolution of the refined map calculated in Cryosparc. (f) Eulerian angle distribution of the particles in the non-uniform refinement. (g) 3DFSC histogram calculated in Cryosparc of the refined map showing angular resolution distribution from 3.0 Å - 3.8 Å.

**Extended Data Fig. 7:**
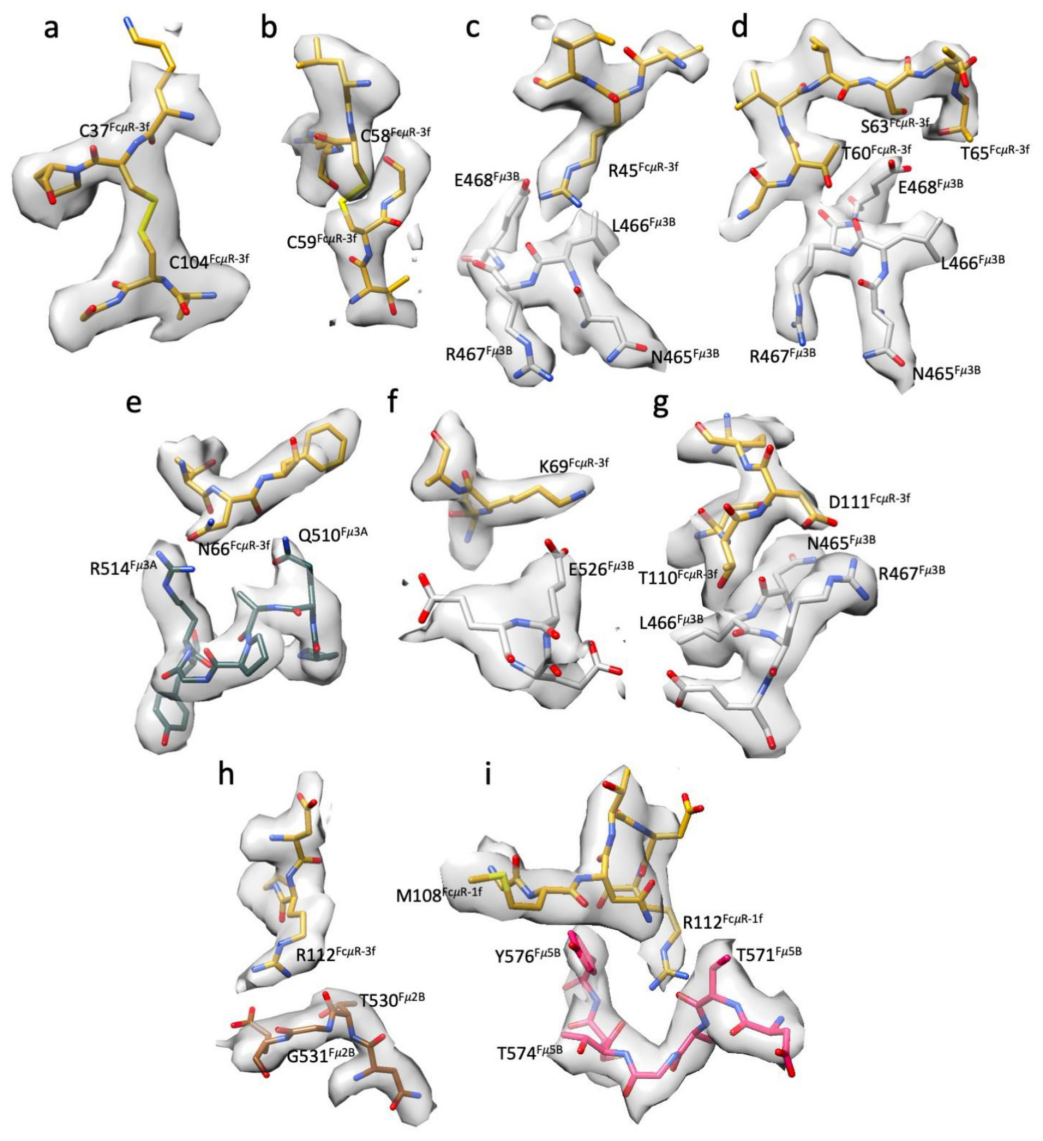
Density maps of key regions at FcμR and FcμR/IgM binding interface. (a-b) The two conserved disulfide bonds in FcμR. (c-g) Densities of the interacting residues on FcμR and Cμ4 domains, corresponding to the interactions shown in Fig. 4b. FcμR in dark yellow, Cμ4-B chain in light grey, and Cμ4-A chain in slate grey. (h) Densities of the residues in CDR3 region of FcμR interacting with the neighbouring Cμ4 domain (in brown), corresponding to Fig. 4c. (i) Densities of the residues in CDR3 regions of FcμR interacting with the tailpiece of Fcμ5 chain (in pink), corresponding to Fig. 4d.

**Fig. 3:**
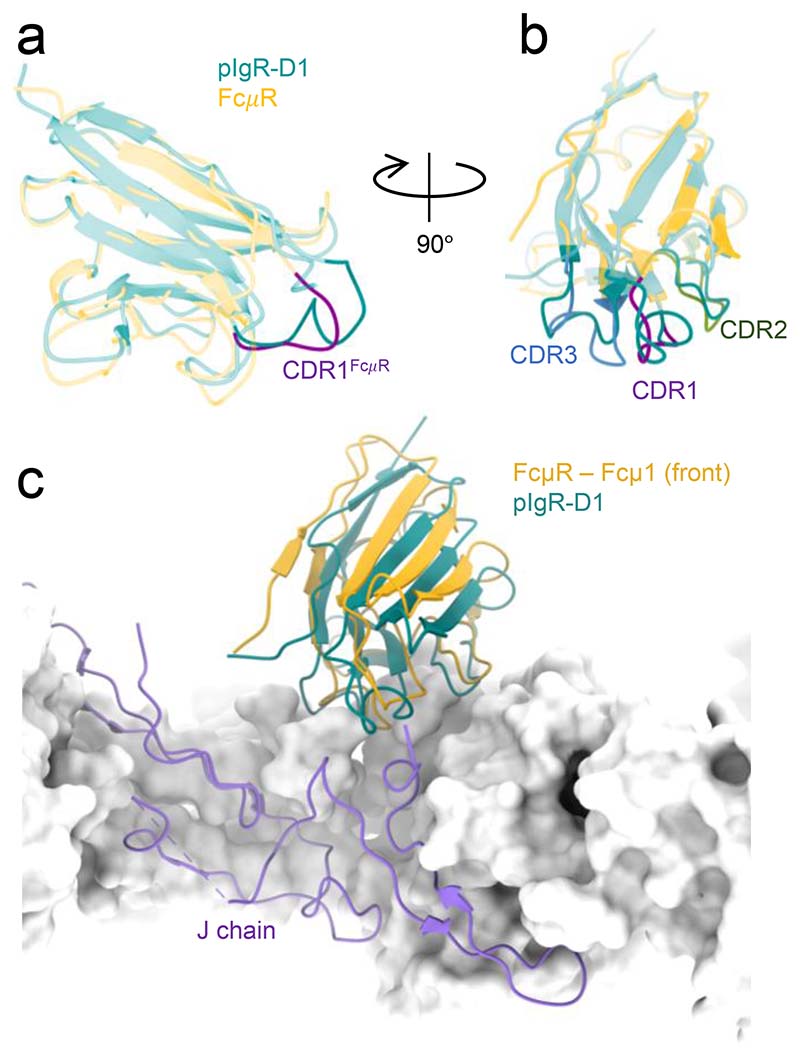
Structure of FcμR Ig-like domain and in comparison to pIgR-D1. (a-b) Superimposed FcμR and pIgR-D1 with the three CDR loops highlighted in FcμR. CDR1, purple; CDR2, green; CDR3, blue. The C*α* root mean squared deviation (RMSD) between the superimposed FcμR Ig-like domain and pIgR-D1 structures (Fig, 3a-b) is 1.639 Å^2^ calculated with 102 aligned residues within the Ig-like domains of the receptors. (c) Overlapped binding sites for pIgR-D1 (cyan) and FcμR at subunit Fcμ1, aligned at IgM, revealing a lifted position of FcμR compared to pIgR-D1.

Two binding sites (position 1f and 3f) which have highest occupancies of FcμR, reached high-resolution (3.5 Å and 3.1 Å) in the cryo-EM maps (Extended Data Fig. 5-7), thus allowing the atomic model interpretation of the FcμR Ig-like domain and the IgM binding interface. At this resolution, we have assigned side-chain locations and can only propose putative bonding arrangements for the interface residues. As anticipated^10,11,17^, the Ig-like domain of FcμR is structurally similar to pIgR-D1 with a root mean squared deviation (RMSD) of 1.64 Å^2^ on C*α* atoms (Fig. 3a-b), with several conserved structural features including two intrachain disulfide bonds (Cys49-Cys58 and Cys37-Cys104), as well as three loops analogous to the complementarity-determining regions (CDR) of immunoglobulin variable domains, which are responsible for engaging IgM.

The FcμR at position 1f overlaps with the single pIgR-D1 binding sites observed in the IgM/pIgR complex^4,6^, but in a slightly lifted position relative to the IgM (Fig. 3c) and with 25% smaller buried surface area (Extended Data Table 1). This difference is likely due to the truncated CDR1 region in FcμR (Fig. 3a). The CDR2 and CDR3 regions of the two receptors are structurally similar (Fig. 3b).

**Fig. 4:**
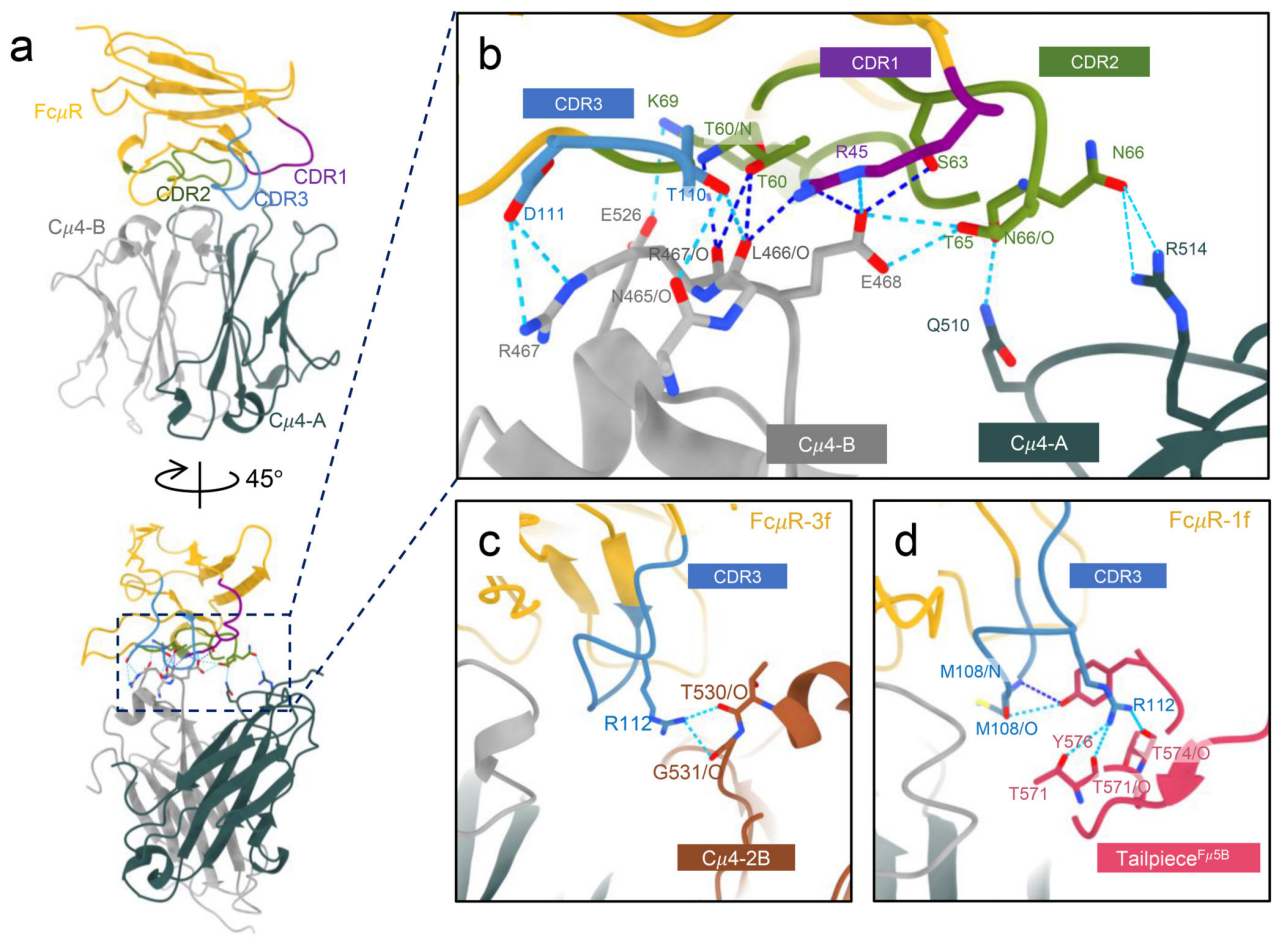
The binding interface between FcμR and IgM. (a) The interactions between the three CDR loops of FcμR and the two Cμ4 domains of the subunit Fcμ3. Cμ4-A in slate grey, Cμ4-B in light grey. (b) Zoom-in view of the region highlighted in the dashed box in (a) showing the interacting residues on the three CDR loops of FcμR and the two Cμ4 domains. The interactions displayed are at subunit Fcμ3 but also shared in subunit Fcμ1. The hydrogen bonds (H-bonds) are represented by dashed lines in blue. Dark blue lines are conserved H-bonds between pIgR-D1 and IgM, and the light blues are unique in FcμR/IgM interface. (c) Interaction of CDR3 of the FcμR at subunit Fcμ3 with the neighbouring Cμ4 domain in IgM subunit Fcμ2 (in brown). (d) Interaction of CDR3 of the FcμR at subunit Fcμ1 with the tailpiece in the B chain of subunit Fcμ5 (in pink). The local resolutions at the binding interfaces shown in (b-d) is around 3 Å (the map densities at individual H-bonds are shown in Extended Data Fig. 7), therefore cautions should be taken when interpreting the interactions at this resolution.

**Fig. 5:**
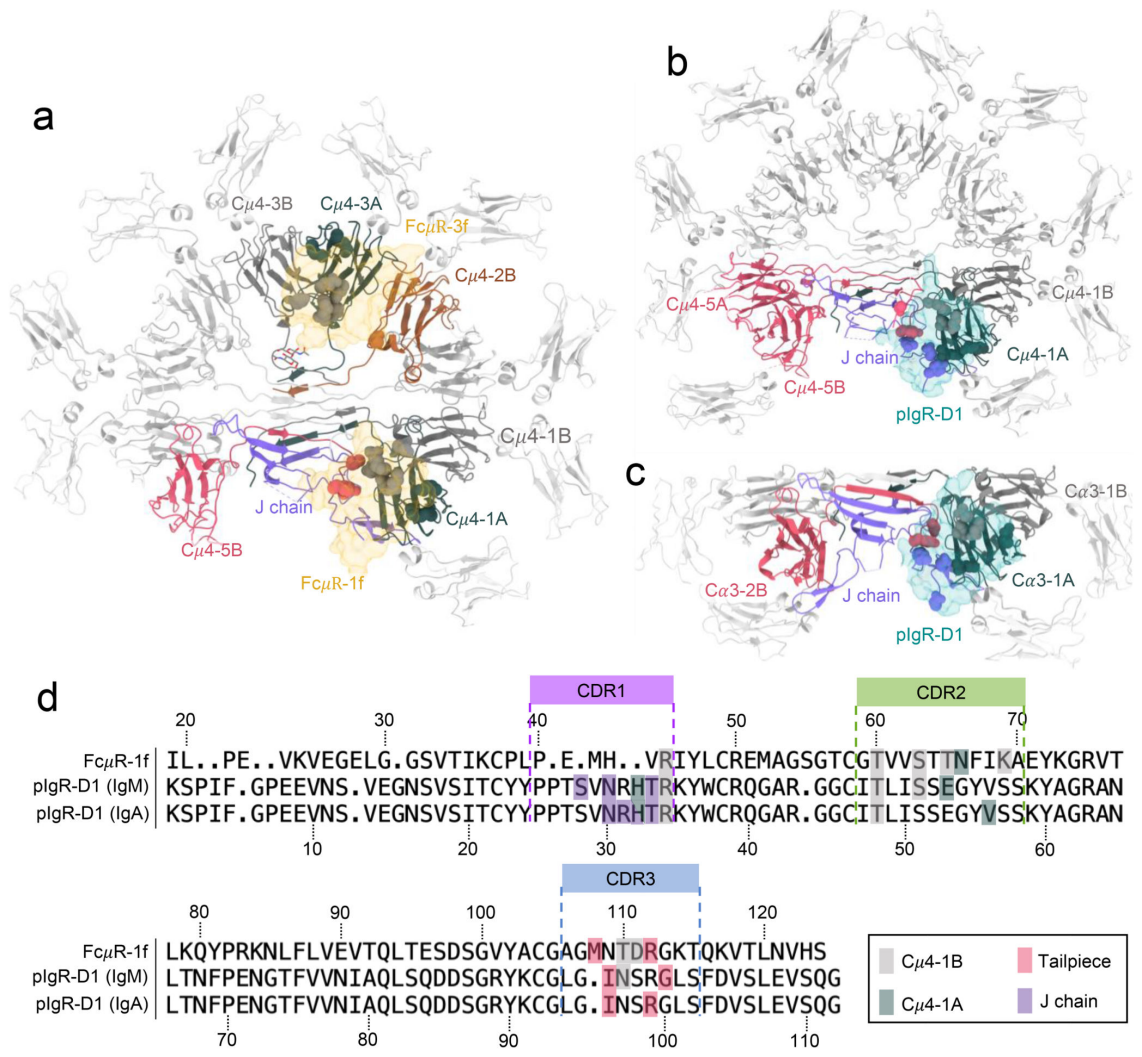
Distributions of interacting residues on the Fc receptors and the targeted immunoglobulin molecules. (a) IgM-Fc core with binding residues for FcμR highlighted in balls at position 1f and 3f. (b) IgM-Fc core with binding residues for pIgR-D1 highlighted in balls. (c) IgA-Fc core with binding residues for pIgR-D1 highlighted in balls. Same colours for chains used in Fig. 4. (d) Structural-based sequence alignment of FcμR at subunit Fcμ1 (first row) and pIgR-D1 (second row) highlighting residues interacting with the indicated regions of IgM. The third row shows pIgR-D1 interactions with IgA.

Due to the quasi six-fold symmetry within the Cμ4 domains in IgM pentamer, the FcμR molecules at position 1f and 3f share almost identical interactions with the two Cμ4 domains (Cμ4-A and Cμ4-B) in subunits Fcμ1 and Fcμ3 respectively (Fig. 4a-b, Fig. 5a). The Cμ4-B chain of the IgM subunit (grey in Fig. 4b) contains a central hub of residues (Asn465-Glu468) for FcμR binding, which interacts with all three CDR loops on FcμR (Fig. 4b) including CDR1 (Arg45), CDR2 (Thr60/Ser63/Thr65) and CDR3 (Thr110/Asp111). The interactions with Arg45, Thr60 and Ser63 are conserved in pIgR-D1/IgM (highlighted by dark blue dotted lines in Fig. 4b). CDR2 of FcμR forms additional interactions including Lys69, which form a hydrogen bond with Glu526 on Cμ4-B chain and Asn66, which contacts neighbouring Cμ4-A chain (Gln510 and Arg514) (Fig. 4b). Gln510 was identified as a binding site for FcμR and IgM by mutagenesis^10^. Asn66 in human FcμR is substituted by a gap in mouse FcμR, which could be one of the reasons for the weaker binding between mouse FcμR/IgM binding^17^.

**Extended Data Fig. 8:**
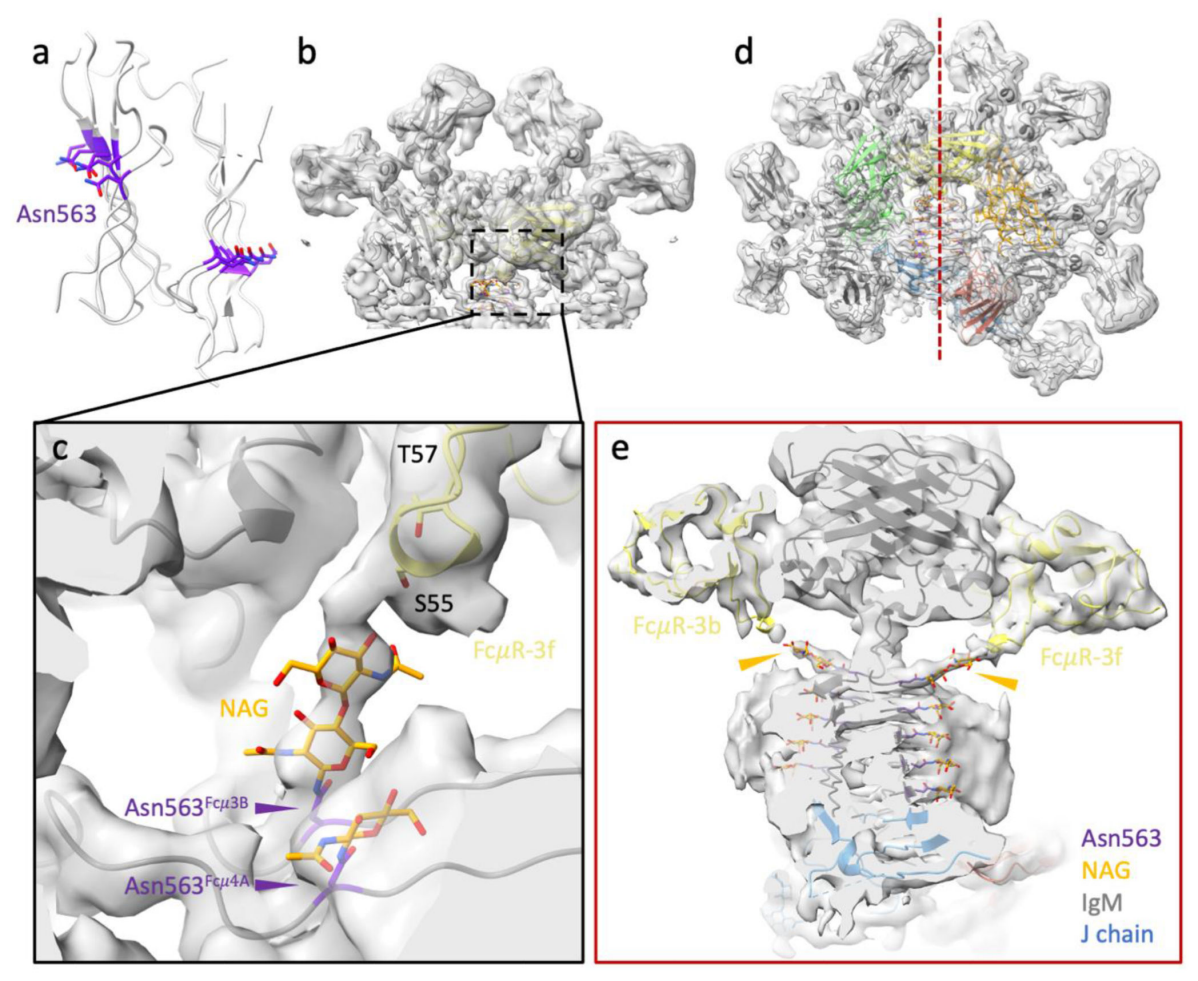
N-linked glycosylation at Asn563 contacting FcμR at subunit Fcμ3. (a) The tailpiece assembly of the IgM pentamer showing ten N-linked glycosylation sites (purple). (b) The map of subunit Fcμ3 (same map as Extended Data Fig. 6a, before postprocessing, map threshold=0.2). (c) Zoom-in view of the N-Acetylglucosamine (NAG) molecules (orange) linking from Asn563 (purple) at the tailpiece of Fcμ3B chain to FcμR-3f (yellow). (d) The overall map of FcμR/IgM-Fc (same map as Extended Data Fig. 3a, before postprocessing, map threshold=0.2). (e) Cross-section of the map in (d) indicated by the red dotted line showing the densities of the two NAG chains (orange arrowheads) extending from Asn563 of the two Fcμ chains (Fcμ3A and Fcμ3B) to the two FcμR molecules at both sides.

In addition to the common features, different interactions between FcμR and binding sites at subunits Fcμ1 and Fcμ3 result from the asymmetrical structure of IgM pentamer. At position 3f, Arg112 in FcμR interacts with two carbonyl groups (Thr530 and Gly531) on the Cμ4-2B domain in the neighbouring IgM subunit (Fig. 4c and Fig. 5a), which is presumably shared by all the FcμR bound at internal subunits (subunits Fcμ2-4). Furthermore, additional contact from the carbohydrate chain extending from Asn563 on the tailpiece towards the FcμR is observed at subunit Fcμ3 (Extended Data Fig. 8). Interestingly, although all ten Asn563 sites are glycosylated, only the carbohydrates at subunit Fcμ3 are involved in FcμR engagement. This is due to the mismatch between the quasi six-fold symmetrical FcμR binding sites and the two-fold symmetrical IgM tailpiece where Asn563 is located, resulting in different relative positions between the glycosylation sites and FcμR molecules at different subunits. This may explain the higher FcμR occupancies at both positions 3f and 3b compared to the adjacent subunits. Nevertheless, it has been found that FcμR can still engage with de-glycosylated IgM and trigger internalisation by the cells^9^. The same residue Arg112 in FcμR at position 1f, on the other hand, interacts with Thr571 and Thr574 at the tailpiece in subunit Fcμ5 (chain B) extended from the other side of the IgM-Fc core (Fig. 4d and Fig. 5a). Met108 in FcμR also forms hydrogen bonds with Tyr576 at the end of the same tailpiece. The interactions with the tailpiece residues may provide extra stabilisation for FcμR, directly reflected in the high occupancy at position 1f. Interestingly, FcμR at position 1f on IgM, although overlapping with the binding site for pIgR-D1, does not interact with the J chain as pIgR-D1 does when binding to either IgM or IgA (Fig. 5b-c), consistent with FcμR binding at other sites where it cannot interact with the J chain.

### Fcμ specificity of FcμR

The interactions observed between the CDR loops in FcμR and the IgM-Fc core provide a structural basis for IgM specificity of FcμR. The CDR1 loop in FcμR is four residues shorter than the same region in pIgR-D1. Only Arg45 at the tip of CDR1 of FcμR is conserved (Arg34 in pIgR-D1), whereas the other key CDR1 residues in pIgR-D1 which have multiple interactions with the J-chain are missing in FcμR. Therefore, FcμR binding on IgM is predominantly stabilised by CDR2 and CDR3 loops. On the other hand, CDR1 contributes significantly to pIgR binding to both Ig isotypes (Fig. 5d), more so for the IgA dimer (pdb id 6UE7) than for IgM pentamer (pdb id 6KXS) based on the percentage of buried surface area involved (35.2% vs 26.5%, Extended Data table 1). Fewer residues on pIgR-D1 in CDR2 and CDR3 regions are involved in interactions with IgA than IgM (four vs six, Fig. 5d). As a result, a truncated CDR1 would have a greater impact on receptor binding to IgA than IgM. In addition, two of the residues on IgM responsible for FcμR binding (Arg514 and Arg467) are not conserved in IgA (Glu363 and Asn362), which would potentially disrupt the interactions at Asn66 (CDR2), Lys69 (CDR2) and Asp111 (CDR3) in FcμR. These differences between the two receptors and Ig isotypes likely account for FcμR specificity for IgM.

## Discussion

We determined the structure of FcμR Ig-like domain at multiple FcμR binding sites on the IgM pentamer, where the Ig-like domain binds to Cμ4 dimers though can make additional interactions with either the adjacent subunit Cμ4 or tailpiece. The subnanomolar binding affinity of pentameric IgM to immobilised FcμR reported here and by others^1^ likely reflects high-avidity binding of IgM to multiple FcμR molecules, the potential for which has been shown in the structures of the complex. Multivalent engagement of IgM facilitates the capture of soluble IgM or IgM immune complexes by FcμR anchored on cell surfaces, leading to FcμR clustering and signalling^18^. Clustering has been found to be essential to induce phosphorylation at the serine and tyrosine residues in the ﻿immunoglobulin tail tyrosine (ITT) motif within the cytoplasmic region of FcμR^1^. The complex provides a structural basis for the observed colocalisation of FcμR and IgM-BCR on membranes of mature B cells that promote B cell survival^19^ and on the trans-Golgi network (TGN) in developing B cells that regulates the transport of IgM-BCR from TGN to the B cell surface^20^.

The stalk region of FcμR, although disordered and largely indiscernible in the cryo-EM map, may indicate flexibility of the stalk region mediating membrane attachment. The stalk of FcμR contains several O-linked glycosylation sites^21^, and its long and flexible nature potentially enhances capture of IgM or IgM immune complexes by surface-anchored FcμR. More structural details of the stalk regions will be required to see if they can interact with the stalk of adjacent FcμR or other molecules.

We describe structural similarities and differences between FcμR Ig domain and pIgR-D1, and one of the FcμR binding sites overlaps with the single IgM binding site observed for D1 in the context of the five-domain secretory component (SC), where D1 is necessary and sufficient for IgA binding^22^. pIgR-D1 alone binds with sub micromolar affinity (K_d_ ˜300 nM) to IgA^23^ at a site similar to the IgM binding site consisting of Cμ4 and the J chain. These similar interactions depend on CDR1 in D1 and are absent in FcμR and therefore explain the specific binding of FcμR to IgM. Comparison of the IgM-Fc core with and without the SC of pIgR bound suggests that SC binding does not induce significant changes in the IgM-Fc core^13^. Because SC only occupies a single site on IgM with the D1 domain and the other four Ig domains (D2-D5) do not occlude the remaining FcμR binding sites, seven positions are available (positions 2f-4f and 2b-5b) for FcμR binding on the IgM/SC complex, so potentially FcμR could work in concert with pIgR in mucosal immunity^24^.

A key common feature of the FcμR binding sites is that only the Cμ4 domains are involved in the interactions. They therefore recognise an invariant region, whereas other parts of IgM, including Cμ3, may vary in conformation^13,15^. Overall, our structures reveal the binding and isotype specificity of FcμR for IgM and promote the understanding of the functions of FcμR in the BCR signalling pathway.

## Methods

### IgM-Fc/Fc*μ*R complex sample preparation

Full-length IgM (myeloma, Jackson Immunoresearch, 009-000-012) was HPLC purified using Superose 10/300 column with running buffer (Tris-HCl buffer 50 mM, 11.5 mM CaCl_2,_ pH 8.1). The IgM sample was then concentrated to around 2 mg/mL and ultrapure trypsin (New England Biolabs, P8101S) with equivalent 4% weight of IgM was added to the sample for proteolysis. The reaction was kept at 56°C for 30 min. The proteolysed sample was then run again on HPLC column again using the same Superose column above with PBS as running buffer to isolate the correct IgM-Fc fragment, and fractions were double-checked by visualising on 3-8% Tris Acetate protein gels. The IgM-Fc sample was diluted to 2*μ*M in PBS. Lyophilized human Fc*μ*R (R&D, Catalog 9494-MU-050) was suspended in PBS to 20 *μ*M.

### Bio-layer interferometry

BLI experiments were performed in PBS buffer and 0.005% Tween 20 on an Octet R8 instrument (Sartorius) operating at 25 °C. The anti-FcμR antibody (Toso Antibody (RR-16)) was purchased from Santa Cruz Biotechnology (cat no. sc-101253). Octet AMC (anti-mouse IgG-Fc capture) biosensors were loaded with anti-FcμR antibody (2 μg/ml) and then FcμR (20 μg/ml) in two subsequent steps. The sensors were then exposed to different concentrations of full-length or proteolysed IgM (0.08-10 nM). Two technical replicates were conducted for each form of IgM.

Association and dissociation curves were recorded for each concentration. Data were analysed as previously described^25^. Apparent equilibrium dissociation constants (K_d_) were determined from the instrument response against IgM concentration using a 1:1 binding model using least squares non-linear regression. In control experiments, sensors with immobilised anti-FcμR antibody (2 μg/ml) were exposed to varying IgM concentrations.

### IgM-Fc/Fc*μ*R complex cryo-EM grid preparation

The proteolysed human IgM-Fc (2 *μ*M) were mixed with the resuspended human Fc*μ*R (20 *μ*M) with the same volume. The solution was incubated at room temperature for 10 min, and then diluted 10-fold just before plunge freezing to final concentrations of 0.1 *μ*M for IgM-Fc and 1 *μ*M for Fc*μ*R. Quantifoil (300 Cu mesh, R2/2) was washed by chloroform, dried in air, and glow discharged (EMITECH K100X) with air (25 mA, 30 s). 4 μl of diluted sample was pipetted to the grid in the environmental chamber of a Vitrobot Mark IV (FEI/Thermo) at 4 °C and 100% humidity. The grid was blotted for 4 s before plunged into liquid ethane kept at liquid nitrogen temperature.

### Cryo-EM data collection

The IgM cryo grids were first screened on Talos Arctica microscope (FEI/Thermo) at 200 kV and the best ones were transferred to a Titan Krios microscope (FEI/Thermo) at 300 kV equipped with a Gatan Imaging Filter (GIF) using EPU software (v 2.11). The slit width of the energy filter was set to 20 eV. 17,835 movies were recorded on a K2 camera in counting mode with a total dose of 50.6 electrons per Å^2^ fractionated over 40 frames (dose rate 5.06 e^-^/Å^2^/s) with a 1.08 Å pixel size and a defocus range between -1.2 to -3.5 μm. 16,713 additional movies were recorded with the same imaging conditions but with the sample stage tilted by 20*°* to increase the particle orientation distribution.

### Cryo-EM data processing

The workflow of the Cryo-EM data processing is shown in Extended Data Fig. 2a. Both non-tilted and tilted movies were imported into Relion (v 3.1)^26^, followed by Relion’s own motion correction and CTF estimation (CTFFIND, v 4.1.13)^27^. For the tilted dataset, patchCTF in CryoSPARC (v 3.2.0)^28^ was also conducted in parallel on the aligned micrographs to obtain a better estimation of the defocus values. 5.9M particles in total were picked by a trained model in CrYOLO (v 1.7.5)^29^ from both datasets and extracted in Relion with a box size of 100 pixels (bin4, pixel size=4.32 Å). The original CTF values of the particles in the tilted dataset were then substituted by the results from patchCTF by using the patchCTF extraction function in CryoSparc, and the particles were re-extracted in Relion with the same box size and binning as the non-tilted particles. The particles were then combined and subjected to 2D classification in CryoSPARC, with 2.08 M particles selected based on the populations and resolutions of the class averages. Typical 2D class averages are shown in Extended Data Fig. 2b. The selected particles were re-extracted again in Relion with a box size of 400 pixels with original pixel size (1.08 Å). The particles were then refined, Bayesian polished, and refined again in Relion (unsharpened map in Extended Data Fig. 2c). The half-map FSC at 0.143 is 3.5 Å (FSC plot in Extended Data Fig. 2d).

To address problems caused by the quasi-two-fold symmetry of IgM pentamer and different occupancies of FcμR binding, particle subtraction was conducted to remove the signal of FcμR from the IgM-Fc core (pink mask in Extended Data Fig. 3a), followed by 3D classification without image alignment to separate the particle subset with best resolution at the IgM-Fc core. Highly resolved J chain is an indicator of good particle alignment. 516,875 particles from the 3D class highlighted in the green box in Extended Data Fig. 3a were selected and reverted to the original particles with all the signals restored. The particles were then 3D refined by non-uniform refinement in Cryosparc with CTF refinement options including beam tilt and per-particle defocus correction. The refined map reveals eight FcμR binding sites on IgM-Fc core with different intensities of FcμR, which sequentially appears when lowering the threshold of the Gaussian-filtered map (Fig. 2a).

The relative intensities of densities at individual FcμR binding sites reflect the occupancy of FcμR at each position, which were quantified by focused 3D classification at individual subunits described in Extended Data Fig. 4. Masks were created for individual subunits for particle subtraction, where only signal inside the masks remained. 3D classification without image alignment was then calculated for each subunit. The percentages of the binding states for each subunit (bound at front, back, both, or neither) are presented in Extended Data Fig. 4 and also summarised in Fig. 2b. The results of the 3D classification were not influenced by and therefore independent from the number (from six to fifteen) of the classes used in the calculations.

To resolve the binding interfaces in atomic detail, focused 3D classifications and refinements were performed at the subunits with highest occupancies of FcμR, i.e., at subunit Fcμ1 and Fcμ3. The 3D classifications started from all the particles (2,084,147) in Extended Data Fig. 2 to preserve as many good particles as possible for further refinement. For subunit Fcμ3, two FcμR molecules are bound at both front and back of the subunit, resulting in a local C2 symmetry, which was implemented in 3D classification and refinement. The workflows of focused classification and refinement for subunit Fcμ1 and Fcμ3 are shown in Extended Data Fig. 5 and Extended Data Fig. 6, respectively. CTF refinement parameters including beam-tilt, and per-particle-defocus corrections are also applied to the non-uniform refinements.

### Model building and refinement

The initial atomic coordinate model of Fc*μ*R was predicted by trRosetta online server^30^, and the cryo-EM model (pdb id 6KXS) was used as the initial model for IgM-Fc core.

For the map refined using the particles selected by the focused 3D classification at subunit Fc*μ*1 (Extended Data Fig. 5), all ten Fcμ chains as well as the J chain in the IgM cryo-EM model (pdb id 6KXS) were included as the initial model. One FcμR was built in the density at the front of subunit Fcμ1. Real space refinement in Phenix (v 1.19.2)^31^ was performed before manually fixing the clashes and outliers in Coot (v 0.9.6)^32^. Iterations between auto- and manual-refinements were conducted for optimisation. N-Acetylglucosamine (NAG) molecules on the four IgM Fcμ chains at Asn563 were built and refined in Coot.

For the map refined using the particles selected by the focused 3D classification at subunit Fc*μ*3 (Extended Data Fig. 6), four Fcμ chains (chain C, D, E, and F) from the IgM-Fc cryo-EM model (pdb id 6KXS) were used, and two predicted FcμR models were added at the front and the back of IgM. The refinements were also performed in Phenix and Coot as described above.

Eight Fc*μ*R models were built into the map refined using the particles selected by the focused 3D classification at the IgM-Fc core (Extended Data Fig. 3a). The two models above were combined and the FcμR model refined at subunit Fcμ3 (models in yellow, Extended Data Fig. 6b) was also duplicated and fitted into the FcμR densities at the other five FcμR positions in subunit Fcμ2, Fcμ4, and Fcμ5. Rigid-body refinement was then performed in Phenix for the recombined model (IgM-Fc core + 8 FcμR).

All three cryo-EM maps were sharpened with corresponding models in LocScale^33^ implemented in CCP-EM software (v 1.6.0)^34^ as shown in Extended Data Fig. 3b, 5b and 6b.

### Map and model validation

A series of validation steps were conducted on the maps and models, shown in panel c to f in Extended Data Fig. 3, 5, and 6. The half-map Fourier shell correlation (FSC) for the three structures indicate 3.6 Å, 3.5 Å and 3.1 Å at 0.143 cut-off and map-model FSC plots show 3.9 Å, 3.6 Å and 3.3 Å at 0.5 cut-off (panel c). Local resolution and 3DFSC (panel d and e) were calculated in Cryosparc after refinement. 3DFSC results shown in Extended Data Fig. 3f and 5f indicate some degree of angular anisotropy, caused by preferred orientation of the particles within the ice (Extended Data Fig. 3e and 5e). Peptide chains were validated in Coot and Phenix and carbohydrates were validated using Privateer^35^ in CCP-EM. The data table for Cryo-EM data collection, processing, and validation statistics are summarised in Table 1. RMSD values are calculated in UCSF Chimera (v 1.13.1)^36^. The figures are made with UCSF Chimera (v 1.13.1) and UCSF ChimeraX (v 1.4)^37^.

## Extended Data

**Extended Data Table 1:** Buried surface areas (BSA) between the individual CDR loops of the receptors and the immunoglobulin binding partner. The CDR regions are defined in the sequence alignment in Fig. 5d.

BSA on receptor (Å^2^)	total	CDR1	CDR2	CDR3	other
Fc*μ*R/IgM (pdb id 8BPF)	926	168.7 (18.2%)	230.9 (24.9%)	459.0 (49.6%)	67.3 (7.3%)
pIgR-D1/IgM (pdb id 6KXS)	1231.1	326.4 (26.5%)	256.3 (20.8%)	486.1 (39.5%)	162.3 (13.2%)
pIgR-D1/IgA (pdb id 6UE7)	1031.2	362.6 (35.2%)	319.3 (31.0%)	326.6 (31.7%)	22.7 (2.2%)

## Supplementary Material

Extended Data Fig. 1

## Figures and Tables

**Table 1:** Cryo-EM data collection, processing, and validation statistics.

	8:1 FcμR/IgM-Fc EMD-16150 PDB 8BPE	FcμR at subunit Fcμ1 EMD-16151 PDB 8BPF	FcμR at subunit Fcμ3 EMD-16152 PDB 8BPG
**Data Acquisition**			
Voltage	300 kV		
Microscope	FEI Titan Krios		
Camera	K2, counting		
Calibrated magnification	46,296		
Electron exposure	50.6 e/Å^2^		
Exposure rate	5.06 e/Å^2^/s		
Number of frames per movie	40		
Energy filter slit width	20 eV		
Automation software	EPU		
Stage tilt	0° and 20°		
Defocus range	-1.2 to -3.5 *μ*m		
Pixel size	1.08 Å		
**Data processing**			
Data processing packages	Relion, CryoSPARC, CrYOLO, CCPEM, LocScale		
Initial particle images	5,923,734		
Symmetry imposed	C1	C1	C2
Final particle images	516,875	539,010	743,566
Half-map FSC (0.143, masked, Å)	3.63	3.52	3.13
Map B factor (Å^2^)	124	124	142
**Model Refinement**			
Initial model used	6KXS, trRosetta predicted Fq*μ*R		
Refinement/validation packages	Phenix, Coot, CCPEM, Privateer		
Map-model FSC (0.5, masked, Å)	3.87	3.59	3.35
Map-model CC			
CC_mask	0.64	0.74	0.73
CC_volume	0.63	0.71	0.67
CC_peaks	0.56	0.63	0.54
CC_box	0.65	0.71	0.62
Model composition			
Non-hydrogen atoms	24203	18594	8652
Protein residues	3104	2370	1106
Ligands	NAG:14	NAG:12	NAG:6
Validation			
MolProbity score	1.80	1.88	1.54
Clashscore	5.41	6.28	3.14
Rotamer outliers (%)	0	0	0
Ramachandran outliers (%)	0	0	0
C*β* outliers (%)	0	0	0
Planarity outlier	0	0	0
Chirality outlier	2	1	2
Bond length outlier	0	0	0
Bond angle outlier	4	5	4

## Data Availability

The structural data that support the findings of this study have been deposited in the Protein Data Bank and EM Data bank. The 8:1 FcμR/IgM-Fc model displayed in Fig. 1 has entry number EMD-16150 and PDB-8BPE. The IgM-Fc core with one FcμR at subunit Fcμ1 has entry number EMD-16151 and PDB-8BPF. The IgM subunit Fcμ3 with two FcμR has entry number EMD-16152 and PDB-8BPG. Source data are provided with this paper.
