# Supplementary material for: Structural basis for Fc receptor recognition of immunoglobulin M
Source: Nat Struct Mol Biol. Author manuscript; Available in PMC 2023 Jul 17. (PMC7614769; doi:10.1038/s41594-023-00985-x)
Supplement: Extended Data Fig. 1 [file EMS174340-supplement-Extended_Data_Fig__1.docx]

**
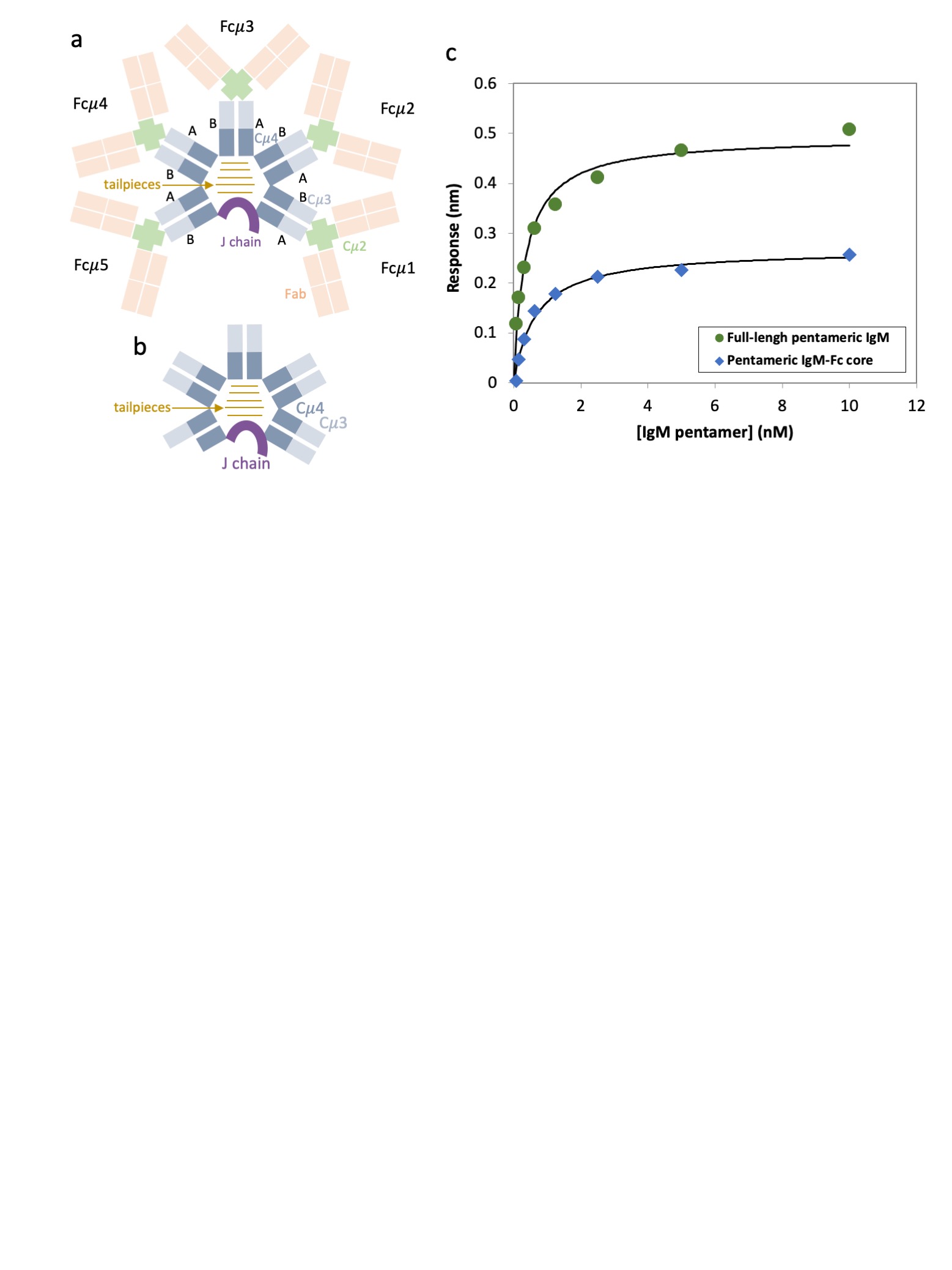
**

**Extended Data Fig. 1. Binding of Full-length IgM and IgM-Fc core to surface-immobilised FcμR.** (a) Structure schematic for full-length IgM. (b) Structure schematic for proteolysed IgM-Fc core. (c) Binding of full-length (green) and proteolysed IgM-Fc core (blue) to FcμR monitored by Bio-Layer Interferometry (BLI). Representative data sets for each form of IgM are shown. Instrument response values are plotted against IgM concentration. Fitting curves are shown as black lines. The apparent equilibrium dissociation constants (K_d_) for full-length IgM and IgM-Fc core are 0.3 ± 0.1 nM and 0.7 ± 0.1 nM respectively. Technical replicates gave values of 0.4 ± 0.1 nM and 0.7 ± 0.1 nM respectively. Raw data for the plot and the technical replicates are provided in the Source Data file.


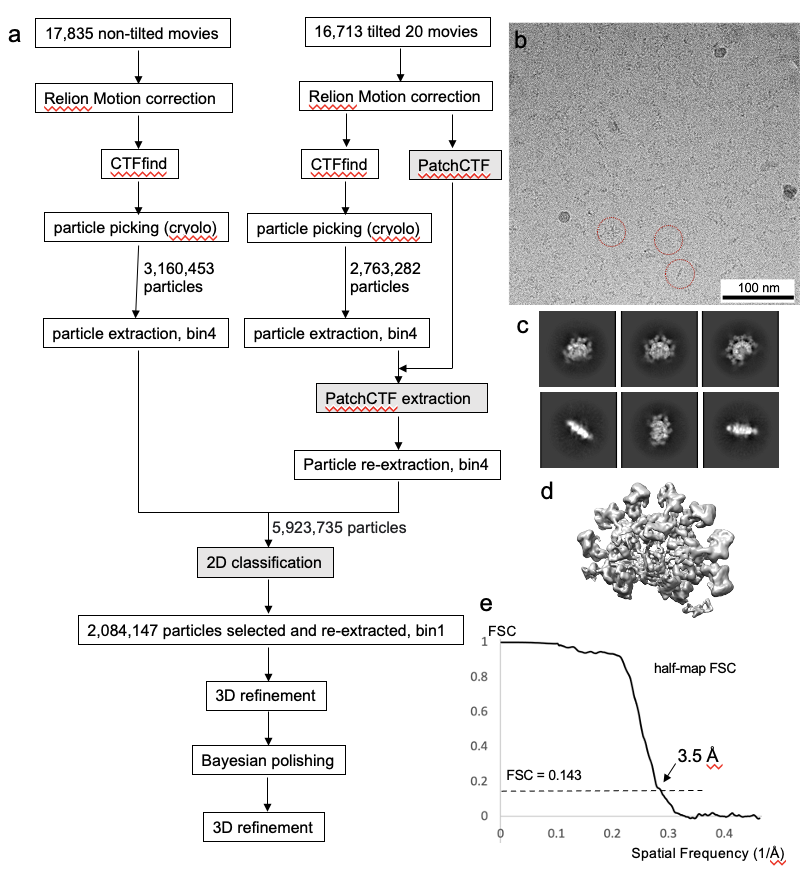


**Extended Data Fig. 2.** **Single particle analysis of FcμR/IgM-Fc.** (a) Flow chart of the data processing for both non-tilted and tilted datasets. Steps are conducted in Relion 3.1 (clear box) or Cryosparc 3.2.0 (grey box). (b) A typical micrograph with three particles highlighted in red dotted circles. (c) Typical 2D classes of the complex. (d) 3D auto-refined map. (e) Half-map Fourier shell correlation (FSC) plot showing 3.5 Å global resolution.


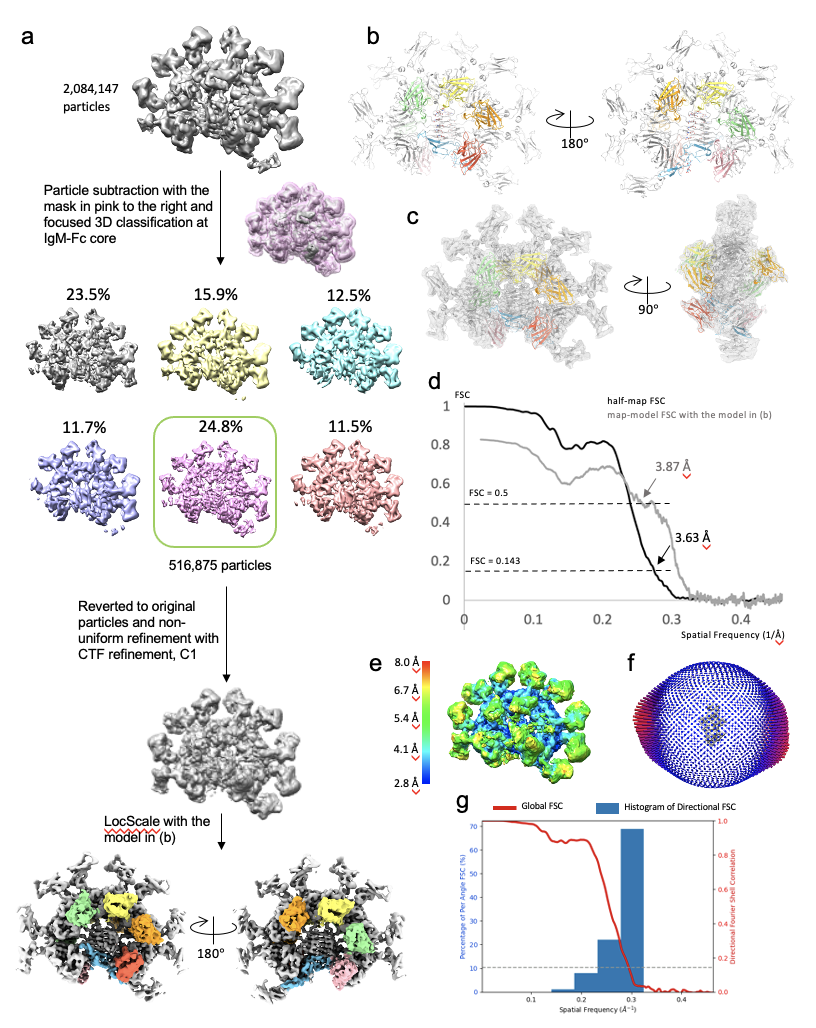


**Extended Data Fig. 3.** **Cryo-EM structure of IgM-Fc/FcμR complex.** (a) Particle subset selection by focused 3D classification at the IgM-Fc core and map refinement. (b) Front and side view of the complex model. IgM in grey, FcμR in rainboow colours. (c) Fitting of model and map shown in (a) and (b). (d) Fourier shell correlation (FSC) with 3.6 Å resolution at 0.143 cut-off and map-model FSC plot showing 3.9 Å resolution at 0.5 cut-off calculated with the model shown in (b) calculated in Phenix. (e) Local resolution of the refined map calculated in Cryosparc. (f) Eulerian angle distribution of the particles in the non-uniform refinement. (g) 3DFSC histogram calculated in Cryosparc of the refined map showing anisotropy between 3.2 Å -5.4 Å.


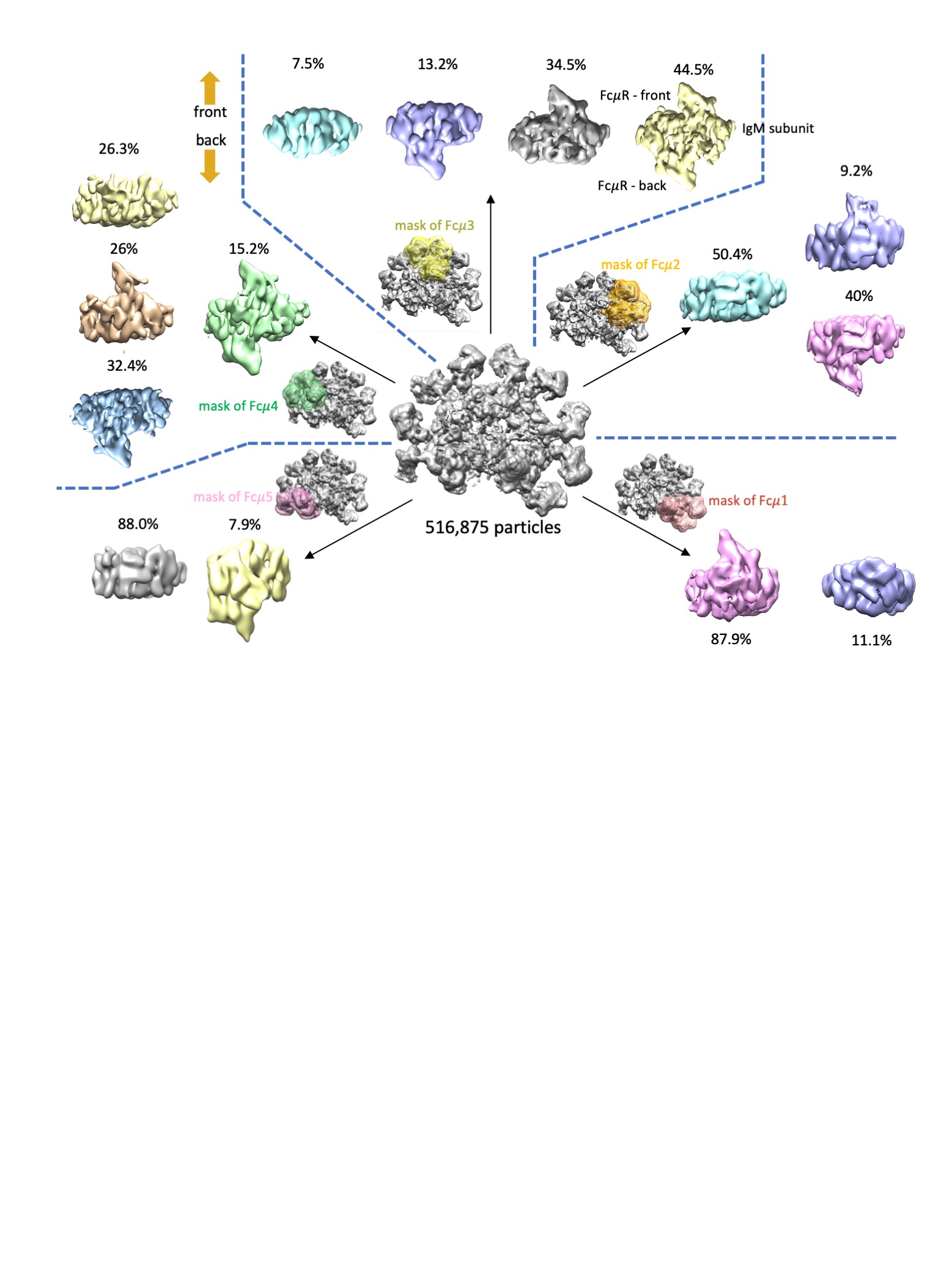


**Extended Data Fig. 4.** **Maps of focused 3D classification at all IgM subunits Fcμ1 to Fcμ5 for quantification of FcμR occupancy at each subunit.** The central EM density is the refined map shown in Extended Data Fig. 3, reconstructed with 516,875 particles. Mask used for focused 3D classification for each subunit (containing Cμ4 dimer, Cμ3 dimer and FcμR) is shown in a specific colour (subunit Fcμ1, red; subunit Fcμ2, orange; subunit Fcμ3, yellow; subunit Fcμ4, green; subunit Fcμ5, pink). The 3D classes show different FcμR binding states (at front, back, both or neither) at each subunit individually. The front and the back of IgM is shown at the top-left of the figure using the same definition described in the main text.

**
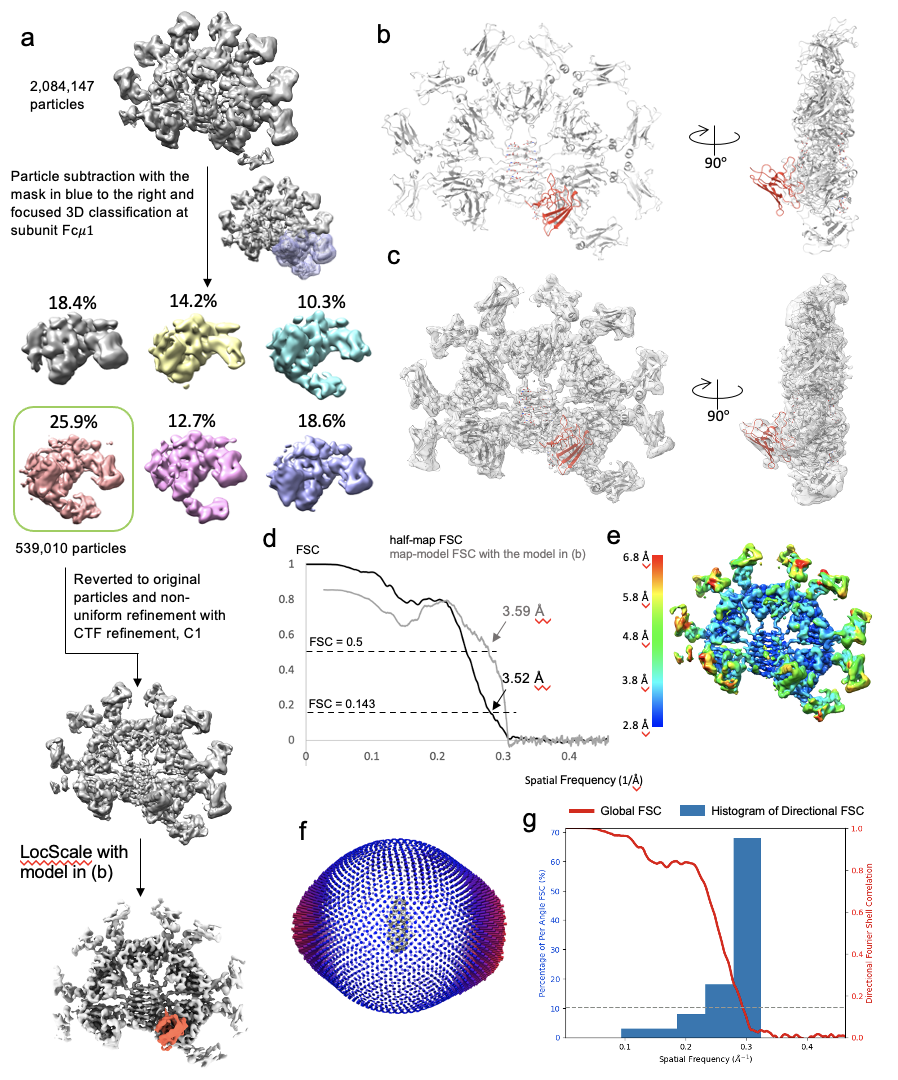
**

**Extended Data Fig. 5. Cryo-EM structure of FcμR/IgM-Fc complex focused on subunit Fcμ1**. (a) Particle subset selection by focused 3D classification at subunit Fcμ1 and map refinement. (b) Front and side view of the complex model. IgM in grey, FcμR in red. (c) Fitting of model and map shown in (a) and (b). (d) Fourier shell correlation (FSC) with 3.5 Å resolution at 0.143 cut-off and map-model FSC plot showing 3.6 Å resolution at 0.5 cut-off calculated in Phenix. (e) Local resolution of the refined map calculated in Cryosparc. (f) Eulerian angle distribution of the particles in the non-uniform refinement. (g) 3DFSC histogram of the refined map calculated in Cryosparc of the refined map showing anisotropy between 3.2 Å -7.6 Å.


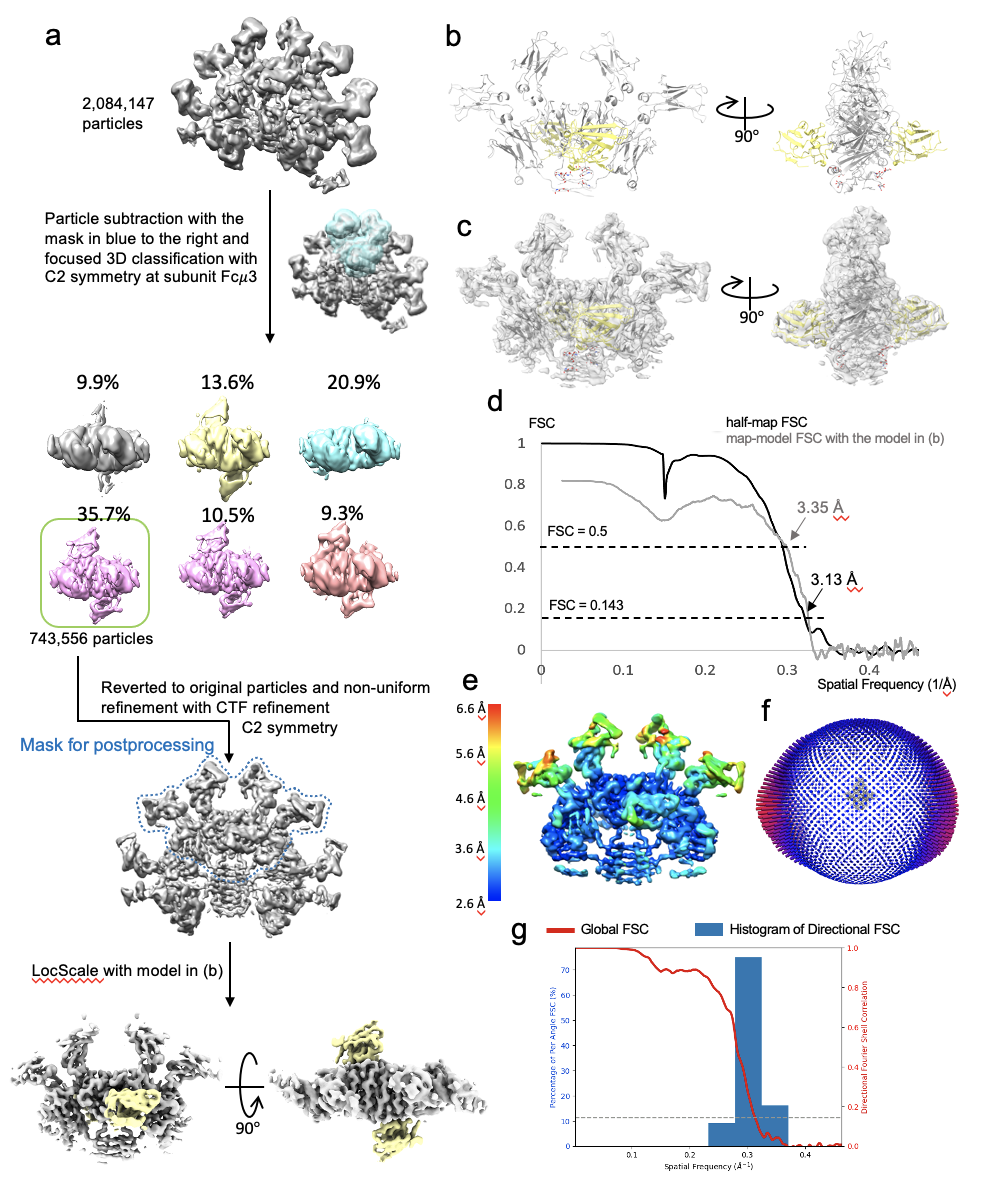


**Extended Data Fig. 6. Cryo-EM structure of FcμR/IgM-Fc complex focused on subunit Fcμ3**. (a) Particle subset selection by focused 3D classification at subunit Fcμ3 and map refinement. (b) Front and side view of the complex model. IgM in grey, FcμR in yellow. (c) Fitting of model and map shown in (a) and (b). (d) Fourier shell correlation (FSC) with 3.1 Å resolution at 0.143 cut-off and map-model FSC plot showing 3.3 Å resolution at 0.5 cut-off calculated in Phenix. (e) Local resolution of the refined map calculated in Cryosparc. (f) Eulerian angle distribution of the particles in the non-uniform refinement. (g) 3DFSC histogram calculated in Cryosparc of the refined map showing angular resolution distribution from 3.0 Å - 3.8 Å.


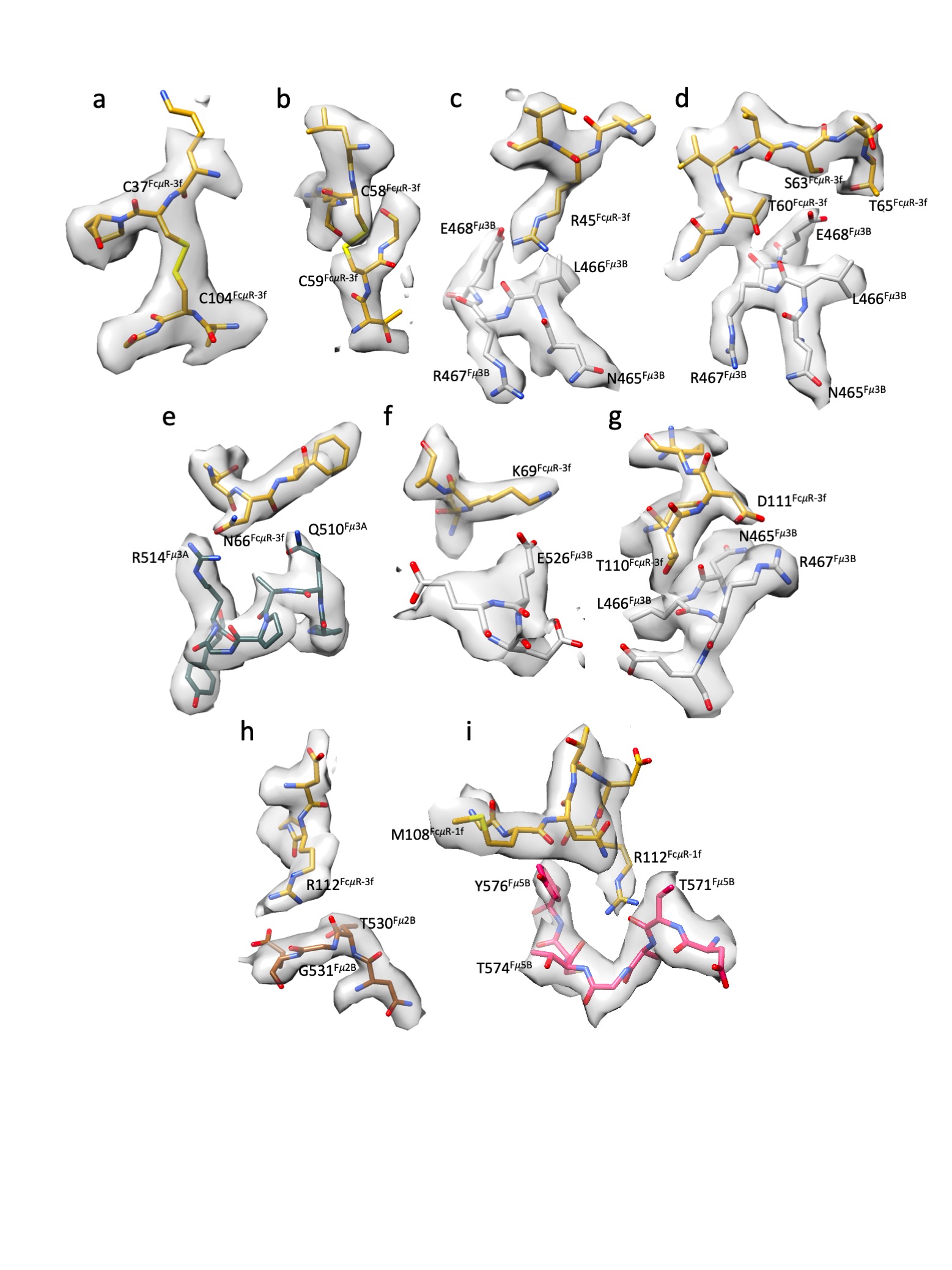


**Extended Data Fig. 7**. **Density maps of key regions at FcμR and FcμR/IgM binding interface.** (a-b) The two conserved disulfide bonds in FcμR. (c-g) Densities of the interacting residues on FcμR and Cμ4 domains, corresponding to the interactions shown in Fig. 4b. FcμR in dark yellow, Cμ4-B chain in light grey, and Cμ4-A chain in slate grey. (h) Densities of the residues in CDR3 region of FcμR interacting with the neighbouring Cμ4 domain (in brown), corresponding to Fig. 4c. (i) Densities of the residues in CDR3 regions of FcμR interacting with the tailpiece of Fcμ5 chain (in pink), corresponding to Fig. 4d.


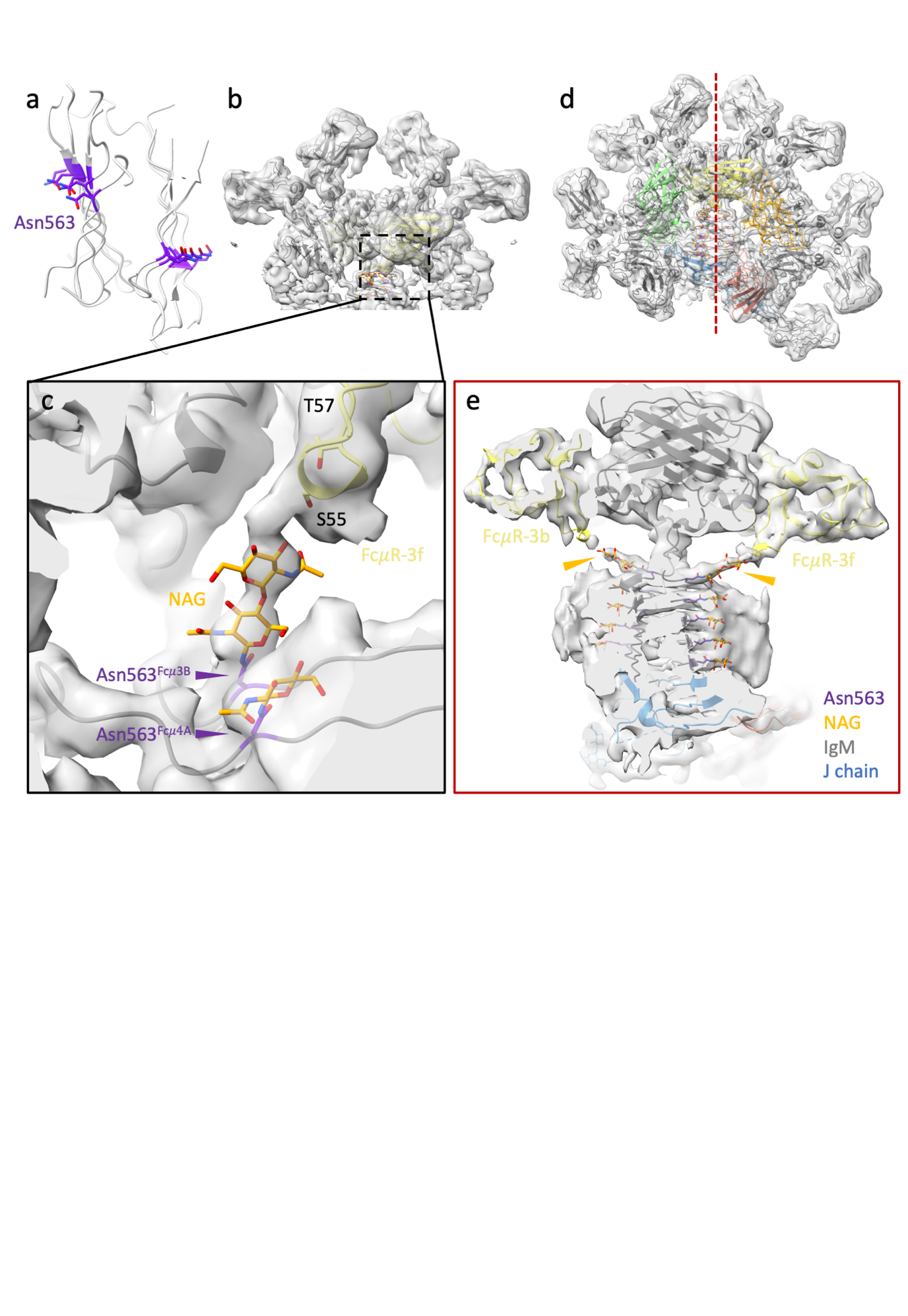


**Extended Data Fig. 8. N-linked glycosylation at Asn563 contacting FcμR at subunit Fcμ3.** (a) The tailpiece assembly of the IgM pentamer showing ten N-linked glycosylation sites (purple). (b) The map of subunit Fcμ3 (same map as Extended Data Fig. 6a, before postprocessing, map threshold=0.2). (c) Zoom-in view of the N-Acetylglucosamine (NAG) molecules (orange) linking from Asn563 (purple) at the tailpiece of Fcμ3B chain to FcμR-3f (yellow). (d) The overall map of FcμR/IgM-Fc (same map as Extended Data Fig. 3a, before postprocessing, map threshold=0.2). (e) Cross-section of the map in (d) indicated by the red dotted line showing the densities of the two NAG chains (orange arrowheads) extending from Asn563 of the two Fcμ chains (Fcμ3A and Fcμ3B) to the two FcμR molecules at both sides.

**Extended Data Table 1. Buried surface areas (BSA) between the individual CDR loops of the receptors and the immunoglobulin binding partner.** The CDR regions are defined in the sequence alignment in Fig. 5d.

| BSA on receptor (Å^2^) | total | CDR1 | CDR2 | CDR3 | other |
| --- | --- | --- | --- | --- | --- |
| Fc$\mu$R/IgM (pdb id 8BPF) | 926 | 168.7 (18.2%) | 230.9 (24.9%) | 459.0 (49.6%) | 67.3 (7.3%) |
| pIgR-D1/IgM (pdb id 6KXS) | 1231.1 | 326.4 (26.5%) | 256.3 (20.8%) | 486.1 (39.5%) | 162.3 (13.2%) |
| pIgR-D1/IgA (pdb id 6UE7) | 1031.2 | 362.6 (35.2%) | 319.3 (31.0%) | 326.6 (31.7%) | 22.7 (2.2%) |
